# The Ras-related gene *ERAS* is involved in human and murine breast cancer

**DOI:** 10.1038/s41598-018-31326-4

**Published:** 2018-08-29

**Authors:** Cristian Suárez-Cabrera, Bárbara de la Peña, Laura L. González, Angustias Page, Mónica Martínez-Fernández, M. Llanos Casanova, Jesús M. Paramio, Alejandro Rojo-Sebastián, Gema Moreno-Bueno, Alicia Maroto, Ángel Ramírez, Manuel Navarro

**Affiliations:** 1 Molecular Oncology Unit, Centro de Investigaciones Energéticas, Medioambientales y Tecnológicas (CIEMAT), 28040 Madrid, Spain; 2 Instituto de Investigación Hospital 12 de Octubre (imas12), Madrid, Spain; 3 Centro de Investigación Biomédica en Red de Cáncer (CIBERONC), 28029 Madrid, Spain; 4 Present Address: Institute of Health and Biomedical Innovation, Queensland University of Technology, Brisbane, Queensland Australia; 5 Present Address: Interdisciplinary Biomedical Research Centre, Nottingham Trent University, Nottingham, UK; 6 Mobile Genomes and Disease Lab, CIMUS - Molecular Medicine and Chronic Diseases Research Centre, Universidade de Santiago de Compostela (USC), Avda Barcelona s/n, Santiago de Compostela, 15706 Spain; 7 Fundación MD Anderson Internacional & MD Anderson Cancer Center, 28033 Madrid, Spain; 8 Departamento de Bioquímica, Universidad Autónoma de Madrid (UAM), Instituto de Investigaciones Biomédicas “Alberto Sols” (CSIC-UAM), IdiPaz, Madrid, 28029 Spain; 9 Department of Pathology, 12 de Octubre University Hospital, Madrid, Spain

## Abstract

Although Ras genes are frequently mutated in human tumors, these mutations are uncommon in breast cancer. However, many breast tumors show evidences of Ras pathway activation. In this manuscript, we have analyzed and characterized mouse mammary tumors generated by random Sleeping Beauty transposon mutagenesis and identify *ERAS* -a member of the RAS family silenced in adult tissues- as a new gene involved in progression and malignancy of breast cancer. Forced expression of *ERAS* in human non-transformed mammary gland cells induces a process of epithelial-to-mesenchymal transition and an increase in stem cells markers; these changes are mediated by miR-200c downregulation. *ERAS* expression in human tumorigenic mammary cells leads to the generation of larger and less differentiated tumors in xenotransplant experiments. Immunohistochemical, RT-qPCR and bioinformatics analysis of human samples show that *ERAS* is aberrantly expressed in 8–10% of breast tumors and this expression is associated with distant metastasis and reduced metastasis-free survival. In summary, our results reveal that inappropriate activation of *ERAS* may be important in the development of a subset of breast tumors. These findings open the possibility of new specific treatments for this subset of ERAS-expressing tumors.

## Introduction

Breast cancer, the second most common cancer in the world and by far the most frequent among women^1^, is a heterogeneous group of diseases. As a consequence, it has been necessary to establish novel classifications at the molecular level in order to group tumors by its biological behavior and prognostic factors such as incidence, survival and response to therapy^2,3^. Traditionally, hormone (estrogen and progesterone) and HER2 receptors status have been used to classify breast tumors. A number of genomic studies have defined several breast cancer intrinsic molecular subtypes, using gene expression profiling^4^. These subtypes (luminal A, luminal B, HER2-enriched, normal-like and basal-like) are associated with different molecular alterations and distinct clinical outcome including therapeutic response^3^. In spite of this, the genes that drive mammary tumorigenesis are only partially known. Recent large scale efforts are starting to identify some of the genes most commonly mutated in breast cancer^5^, but results so far suggest that human breast tumors are very complex, and their development could be triggered by a variety of molecular mechanisms in different individuals. The existence of many low-frequency cancer driver genes that coexist with numerous “passenger” mutations in breast tumors makes their identification by large scale data analysis a daunting task^5^. In addition, genes which are aberrantly activated, but not mutated, are difficult to detect. In this particular concern, the Sleeping Beauty transposon system^6^ is a powerful tool for the identification of cancer driver genes, with an extended history of successfully identified cancer genes in many tumor types^7^. We and others have used this technology to identify genes that drive breast cancer development^8,9^.

The Ras family of small GTPases is an ample group of proteins that exhibit marked amino acid conservation and that share various downstream effectors through which they transmit signals^10^. Although the classical Ras genes (*H-RAS*, *K-RAS4A*, *K-RAS4B* and *N-RAS*) are among the most frequently mutated in human cancer, these mutations are unusual in breast cancer, being found in less than 1% of all cases, according to the catalogue of somatic mutations in cancer (COSMIC)^11^. Nevertheless, the Ras pathway is significantly activated in a number of human breast tumors, particularly in the triple negative group^12^. So, it is possible that other members of the Ras family play a role in progression and malignancy of breast cancer: although the role in cancer of the classical Ras proteins has been studied extensively, the oncogenic potential of other members of the family is less known. And besides mutation in Ras genes, activation of Ras signaling can be carried out by other means, such as inactivation of negative regulators of the Ras pathway (e.g. RasGAPs), as recently shown by us and others^9,13,14^.

In order to identify new driver genes in breast cancer, we generated mammary tumors in transgenic mice bearing multiple copies of a mutagenic Sleeping Beauty (SB) transposon mobilized in mammary glands. Analysis of the transposon insertion sites in these tumors resulted in the identification of two RasGAP genes (*Nf1* and *Rasa1*) as two of the genes most frequently mutated in these mammary tumors, and we established that loss of these genes is a common event in human triple negative breast cancer, thus confirming the implication of the Ras pathway in breast cancer^9^. In addition, some murine mammary tumors bore insertions in the Ras family gene *Eras* (Embryonic stem (ES) cell-expressed Ras). Remarkably, and at difference to all other Ras proteins, ERAS is constitutively active, being insensitive to RasGAP activity. In mice, this gene has an important growth-promoting role during early embryonic development, but its expression is undetectable in differentiated ES cells and adult tissues^15,16^. Given its constitutive activation, aberrant expression of ERAS in adult tissue would have a similar effect to Ras mutation^15^. In this work, we identify *Eras* as a driver gene for murine mammary tumors, report for the first time the expression of ERAS in human breast tumors and identify the mechanisms by which ERAS confers epithelial-to-mesenchymal transition (EMT) and stem cell-like features to human epithelial mammary gland cells.

## Results

### SB/T2 mice develop mammary tumors expressing ERAS

**Figure 1:**
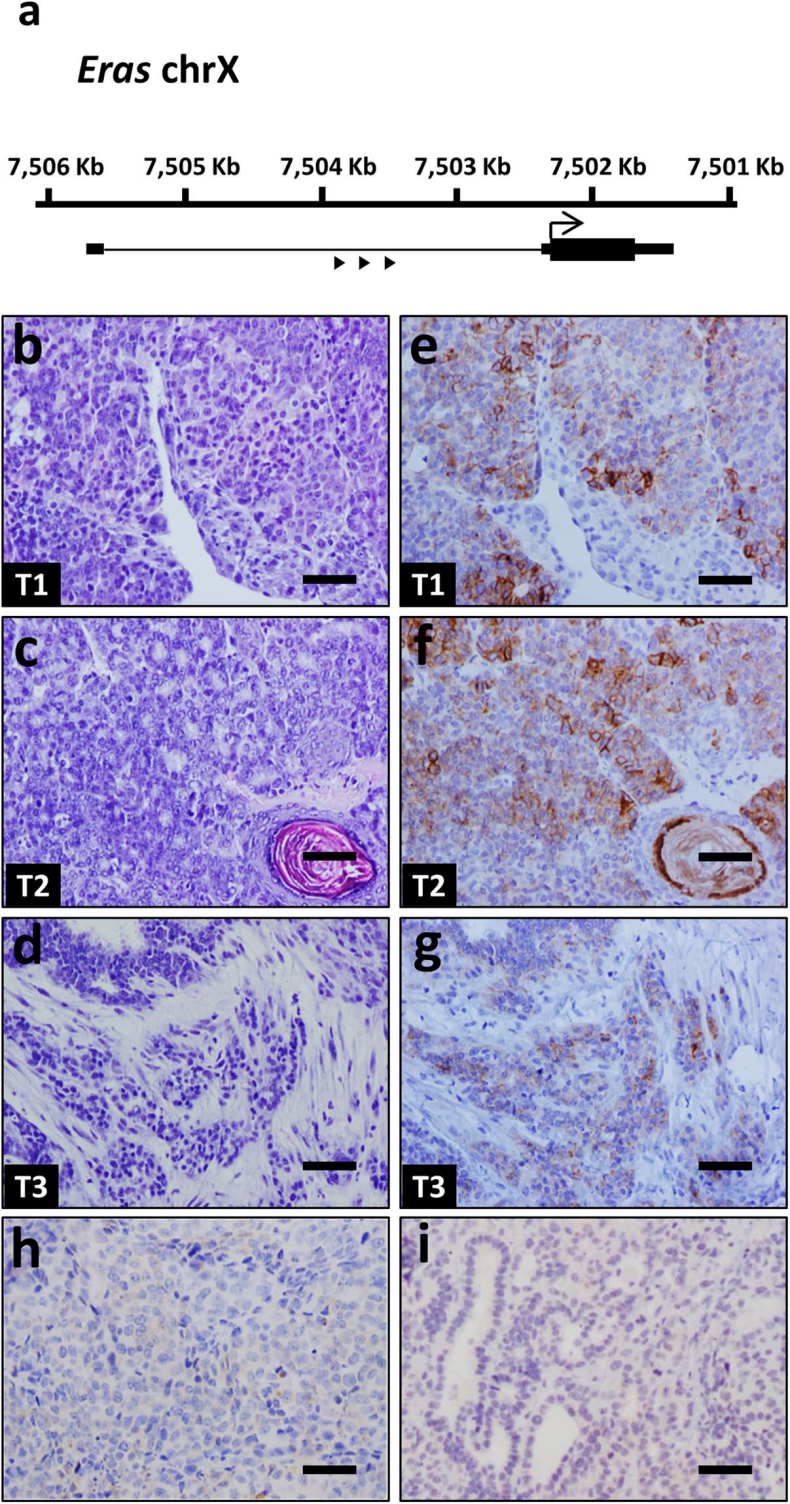
*Eras* expression in mammary gland tumors with activating transposon insertions in *Eras* locus. (**a**) Gene structure of the murine *Eras* gene showing both the localization and orientation (arrowheads) of the transposon insertions into the first intron of *Eras*. (**b**–**d**) Hematoxylin and eosin staining of murine mammary gland tumors with transposon insertion in the *Eras* gene. Image b corresponds to alveolar-papillary carcinoma, image c is an adenosquamous carcinoma, and image d is a tubular-papillary carcinoma with an important myoepithelial cell component. (**e**–**g**) Immunohistochemistry showing ERAS expression (antibody D5G5J) in the tumors shown in images (**b**–**d**) respectively. (**h**,**i**) Representative examples of lack of ERAS staining in tumors without insertion in *Eras*. Bar, 50 μm. Tumor identifications (T1, T2 and T3) are indicated in the lower left corner.

We generated double transgenic mice bearing both a concatemer of T2Onc2 mutagenic transposons and the SB11 transposase under the control of the keratin K5 promoter^9,17^. These mice developed mammary tumors, more frequently in a p53+/− genetic background. We determined by Illumina sequencing their transposon integration sites^9,17^; interestingly, three of 37 mammary tumors (one from p53+/− and two from p53 wt mice) had transposon insertions in the *Eras* gene, suggesting that this event could be important for the development of murine mammary tumors. All three insertions were located in the only intron of this gene, 5′ upstream of the start codon, and all transposon copies were integrated in the same direction as the *Eras* gene, indicating that the result of transposon integration would be transcriptional activation of a full-length *Eras* from the transposon promoter (Fig. 1a and Supplementary Tables SI and SII). Classification based on histological features revealed that these tumors were an alveolar-papillary carcinoma (Fig. 1b), an adenosquamous carcinoma (Fig. 1c) and a tubular-papillary carcinoma (Fig. 1d). Expression of ERAS exclusively in these tumors was confirmed by immunohistochemistry (Fig. 1e–i). ERAS is a constitutively active RAS protein that is not expressed in adult tissues, and given that it seemed to act as a tumor driver in mouse, we decided to study the effect of the expression of *ERAS* in human mammary gland cells and its relation to breast cancer.

### *ERAS* expression induces morphological and proliferative changes to non-tumoral human mammary cells

**Figure 2:**
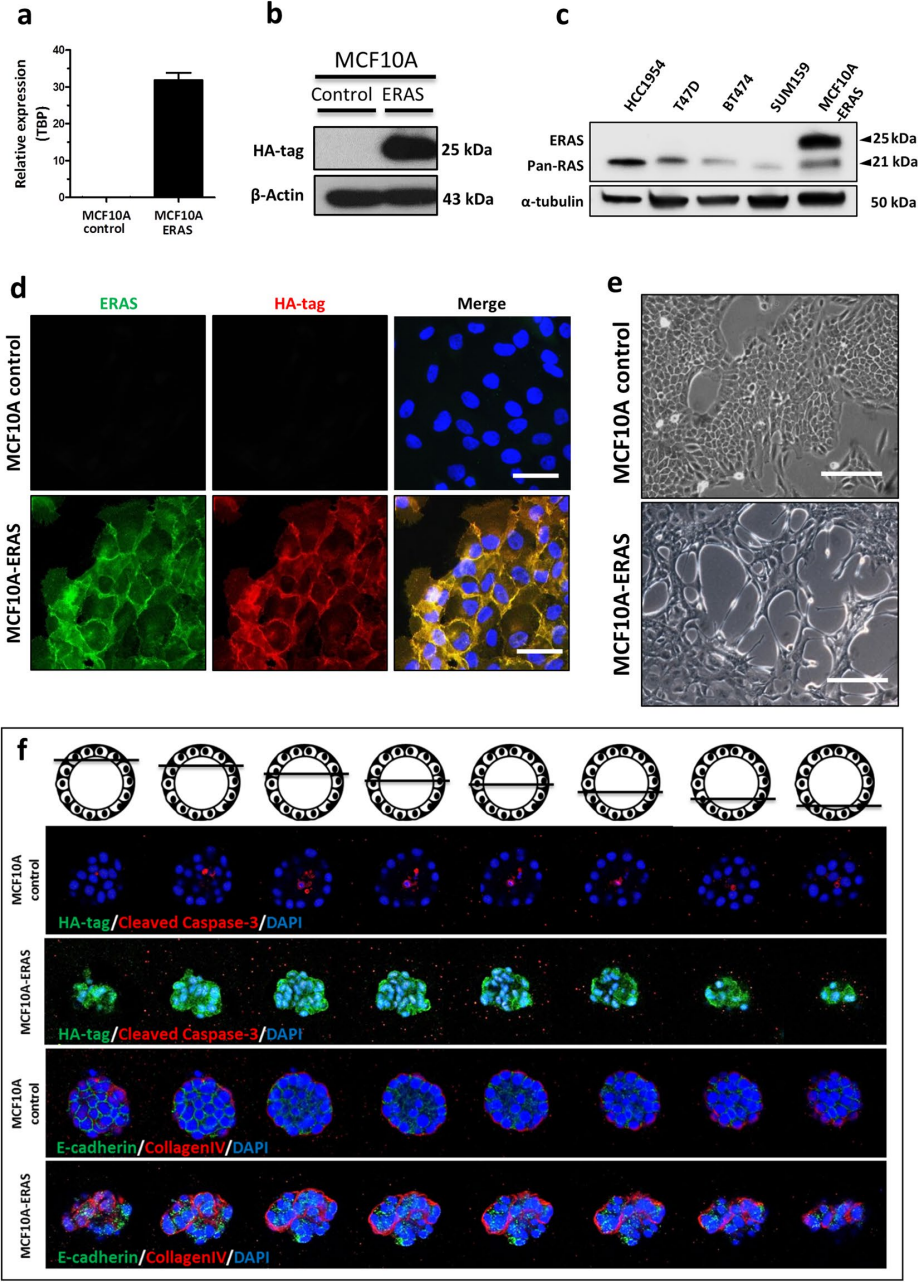
Forced expression of *ERAS* transforms MCF10A cells. Quantification of (**a**) mRNA and (**b**) protein expression of ERAS in transfected MCF10A cells. Relative values in 2a represent the mean ± SD of three different experiments and were normalized with *TBP* expression. (**c**) Western blot showing different mobility for ERAS and other Ras proteins in several human mammary cell lines. The membrane was successively hybridized with the N-20 antibody specific for ERAS (upper band) and the 18/A panRas antibody (lower band). (**d**) Analysis by immunofluorescence showing the localization of ERAS protein in ERAS-transfected cells (lower row). Untransfected cells are shown in the upper row. Note that the signals for HA-tag (red) and ERAS (N20, green) antibodies overlap. DAPI staining is shown in blue. Bar, 50 μm. (**e**) Morphological changes of MCF10A expressing ERAS with respect to control cells; MCF10A-ERAS cells present numerous prolongations and loss of adhesion to substrate and between cells. Bar, 200 μm. (**f**) Disruption of normal formation of mammary acini in three-dimensional culture of ERAS-expressing MCF10A cells. The loss of the spherical and hollow morphology is shown through a series of z-stack confocal microscopy images. HA-tag antibody was used to show expression of *ERAS*, and cleaved caspase-3, E-cadherin, and collagen-IV antibodies were used to visualize apoptosis into acini, junction between cells, and basement membrane, respectively. All experiments were performed in duplicate, typical results are shown. Uncropped blots for b and c are presented in Supplementary Fig. S6.

We cloned the human *ERAS* gene under the control of the CAG promoter^18^ and forced its expression in MCF10A cells, an immortalized non-malignant mammary epithelial cell line which is often used as a model of normal human mammary gland, as it is able to mimic *in vitro* several aspects of mammary development^19^ (Fig. 2a,b). ERAS is larger than the rest of Ras proteins, so it could be easily identified in Western blots (Fig. 2c). Immunofluorescence using ERAS and HA antibodies confirmed that ERAS was mainly located at the cytoplasmic membrane, and also in the cytoplasm and other membranous organelles (Fig. 2d). Expression of *ERAS* induced a sharp phenotypic change in MCF10A cells, developing abundant cytoplasmic prolongations, spindle-like morphology and loss of cell-substrate and cell-cell adhesions (Fig. 2e).

When cultured on a matrigel surface, MCF10A cells form acini-like mammospheres, which recapitulate numerous features of glandular architecture *in vivo*, including the reconstitution of a basement membrane expressing collagen IV and laminin V, and the formation by selective apoptosis of a hollow lumen surrounded by polarized epithelial cells^20,21^. In contrast with control cells, MCF10A-ERAS cells on matrigel formed aberrant irregular non-spherical and non-hollow structures (Fig. 2f). Cleaved caspase-3 was found inside acini from control cells, but not in MCF10A-ERAS acini, indicating lack of apoptosis (Fig. 2f). In the MCF10A-ERAS acini, collagen IV location was also aberrant, being partially lost in the external surface and abnormally present inside the acini, indicating that ERAS-expressing MCF10A cells are unable to develop an external and complete basement membrane. These acini also presented mislocalized and decreased levels of the adherens junction protein E-cadherin, with the subsequent loss of adhesion between cells (Fig. 2f). These results show that *ERAS* expression in MCF10A cells interferes with acinar morphogenesis, resulting in deregulation of polarization, inhibition of apoptosis and loss of adherens junctions.

**Figure 3:**
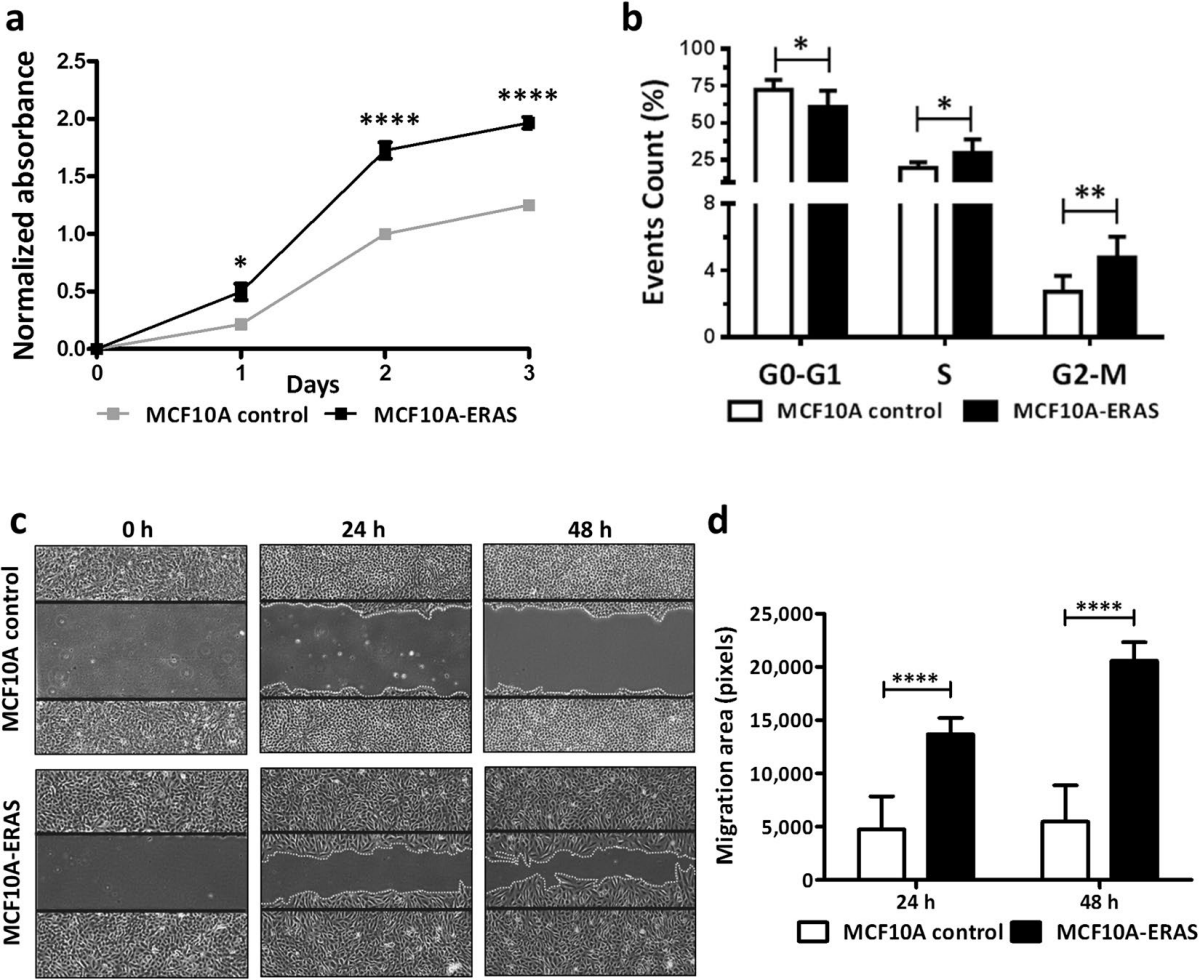
Increased proliferation and migration in MCF10A cells expressing ERAS. (**a**) Representative growth curves of MCF10A control and MCF10A-ERAS cells. Proliferation was measured using XTT proliferation assay. Data represent the means ± SEM of three independent experiments. (**b**) Analysis of the phase distribution of the cell cycle in cells growing at high confluence (85–90%) by flow cytometry in three independent experiments. Note the difference in the scale between the upper and lower parts of the graph. (**c**) Representative images of an *in vitro* wound-healing assay at 0, 24 and 48 hours post-wounding. (**d**) Quantification of the migration area after wounding. Four wounds were analyzed for each cell type in three independent experiments. Migration areas were measured with ImageJ software. In b and d, data represent the means ± SD. Asterisks show significant differences (*p < 0.05; **p < 0.01 and ****p < 0.0001).

ERAS-expressing MCF10A cells also presented a higher proliferation rate than control cells (Fig. 3a). Cell cycle analysis by flow cytometry showed that near-confluent (85–95%) MCF10A-ERAS cells presented an increase in S and G2/M phases (p < 0.05 and p < 0.01, respectively) and a decrease in G0-G1 phase (p < 0.05) compared to control cells (Fig. 3b), suggesting that ERAS expression is enough to overcome growth arrest by contact inhibition in confluent MCF10A cells. MCF10A-ERAS cells also presented a higher migratory capacity than control cells at 24 or 48 hours after wounding (p < 0.0001, Fig. 3c,d).

### *ERAS* induces epithelial-mesenchymal transition and stem cell-like features

**Figure 4:**
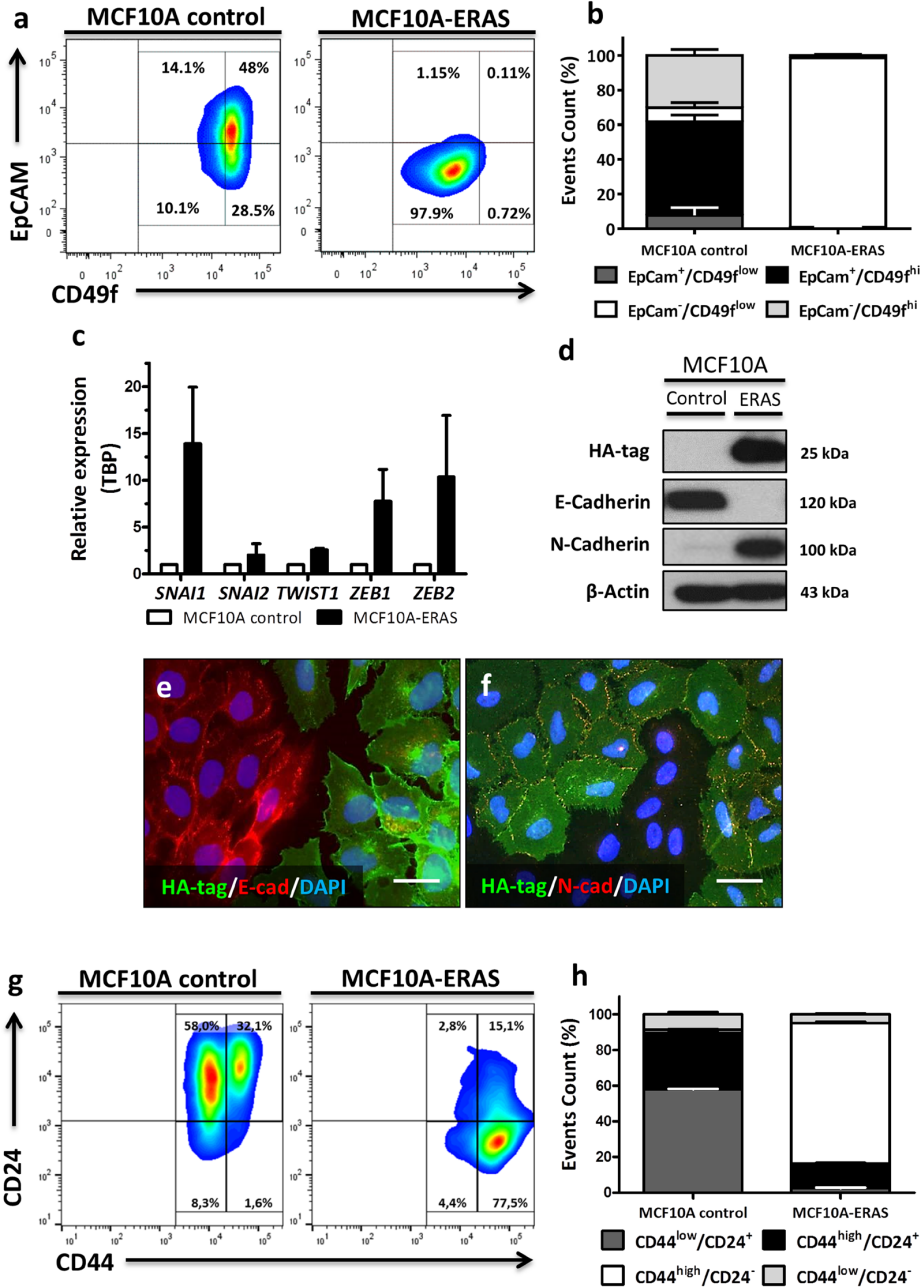
Induction of EMT and stem cell-like features by ERAS expression in mammary gland cells. (**a**) Contour plots of flow cytometry analysis showing EpCAM and CD49f (integrin α6) immunostaining for each cell type. (**b**) Quantification of data shown in a. MCF10A control cells present both EpCAM^+^/CD49f^+high^ and EpCAM^−^/CD49f^+low^ subpopulations, showing mainly an epithelial phenotype, whereas almost all MCF10A-ERAS cells were EpCAM^−^/CD49f^low^ (p < 0.0001). Data represent the means ± SD from three independent experiments. (**c**) Gene-expression levels of transcriptional factors measured by RT-qPCR. Values represent the mean ± SD from three different experiments and were normalized by *TBP* expression and represented in respect to control cells. (**d**) Western blot analysis showing the switch between E-cadherin and N-cadherin in mammary gland cells expressing ERAS. Uncropped blots are presented in Supplementary Fig. S6. (**e**,**f**) Immunofluorescences of HA-tag, E-cadherin and N-cadherin in co-culture of MCF10A control and MCF10A-ERAS cells. Bar, 20 μm (**g**) Representative contour plots of flow cytometry showing CD44 and CD24 immunostaining for each cell type. (**h**) Quantification of data shown in G. CD44^high^/CD24^−^ subpopulation is 1–2% in control cells, however this subpopulation is majority (~80%) in MCF10A-ERAS cells (p < 0.0001). Data represent the means ± SD from three independent experiments.

EMT is an important process in cancer by which epithelial cells lose their polarity and cell-cell contacts, and gain migratory and invasive properties to become mesenchymal cells^22^; in this process, the expression of epithelial genes is repressed, and mesenchymal genes are activated. In order to study in detail this process, we characterized the epithelial and mesenchymal phenotypes of ERAS-expressing MCF10A cells by flow cytometry using specific antibodies for epithelial cell adhesion molecule (EpCAM) and integrin α6 (CD49f) cell surface markers. According to these markers, MCF10A cells include two main subpopulations, whose proportions oscillate in a confluence-dependent manner: whereas EpCAM^+^/CD49f^high^ cells display an epithelial morphology, EpCAM^−^/CD49f^med/low^ cells show a mesenchymal morphology^23,24^. As expected, MCF10A control cells presented both epithelial and mesenchymal subpopulations, being EpCAM^+^/CD49f^high^ cells the most common population (about 50%). In contrast, almost all MCF10A-ERAS cells were EpCAM^−^/CD49f^low^ (p < 0.0001), indicating a strong shift towards the mesenchymal phenotype (Fig. 4a,b).

Multiple transcription factors induce EMT, including ZEB1, ZEB2, TWIST1, SNAI1 (Snail) and SNAI2 (Slug)^22^. These factors suppress the expression of E-cadherin, a major constituent of the adherens junctions, as well as other epithelial markers (such as p120-catenin or β-catenin), but also enhance the expression of mesenchymal genes (such as N-cadherin, vimentin or fibronectin)^25–28^. Gene expression of *SNAI1*, *SNAI2*, *TWIST1*, *ZEB1* and *ZEB2* was analyzed by quantitative RT-qPCR; all of them were augmented in MCF10A-ERAS cells (Fig. 4c). Accordingly, a loss of E-cadherin and a gain of N-cadherin associated to expression of ERAS were observed by western blot (Fig. 4d). Immunofluorescence analyses of co-cultured MCF10A control and ERAS cells confirmed these results (Fig. 4e,f) and showed that other adhesion molecules such as p120-catenin, β-catenin, integrin α6 and β1 were also downregulated (Supplementary Fig. S1a; see also Fig. 4a for integrin α6 expression). In summary, all these results indicate that MCF10A cells undergo a sharp EMT process upon ERAS expression.

Several studies suggest that cells that undergo EMT may also be cells with stem-like properties, with migratory and invasive capabilities associated with metastatic competence^22,26,29,30^. Breast cancer initiating cells are CD44^high^/CD24^−/low^ cells, which retain tumorigenic activity and also display stem cell-like properties, being responsible for cancer progression and metastasis^26,31,32^. In order to determine the role of *ERAS* in inducing stem-like properties in breast epithelial cells, analyses of CD44 and CD24 cell surface markers by flow cytometry were carried out. Forced ERAS expression in MCF10A cells resulted in a tremendous increase (from 1 to 80%) in the CD44^high^/CD24^−^ population, confirming the acquisition of stem-like properties in these cells (Fig. 4g,h).

Self-renewing activity, CD44^high^/CD24^−/low^ cell population and the inability to form differentiated mammospheres have been associated with the loss of lineage-specific markers such as keratins^33,34^. We checked luminal epithelial and myoepithelial keratins such as K8, K18, K5 and K14 in MCF10A-ERAS cells and we observed a reduced expression of all these markers compared to control cells (Supplementary Fig. S1b).

Collectively, these results suggest that *ERAS* expression in human non-tumorigenic mammary cells leads to a marked EMT process and increases the number of cells expressing markers characteristic of mammary stem and tumor cells.

### Understanding *ERAS* signaling pathway in mammary gland cells

**Figure 5:**
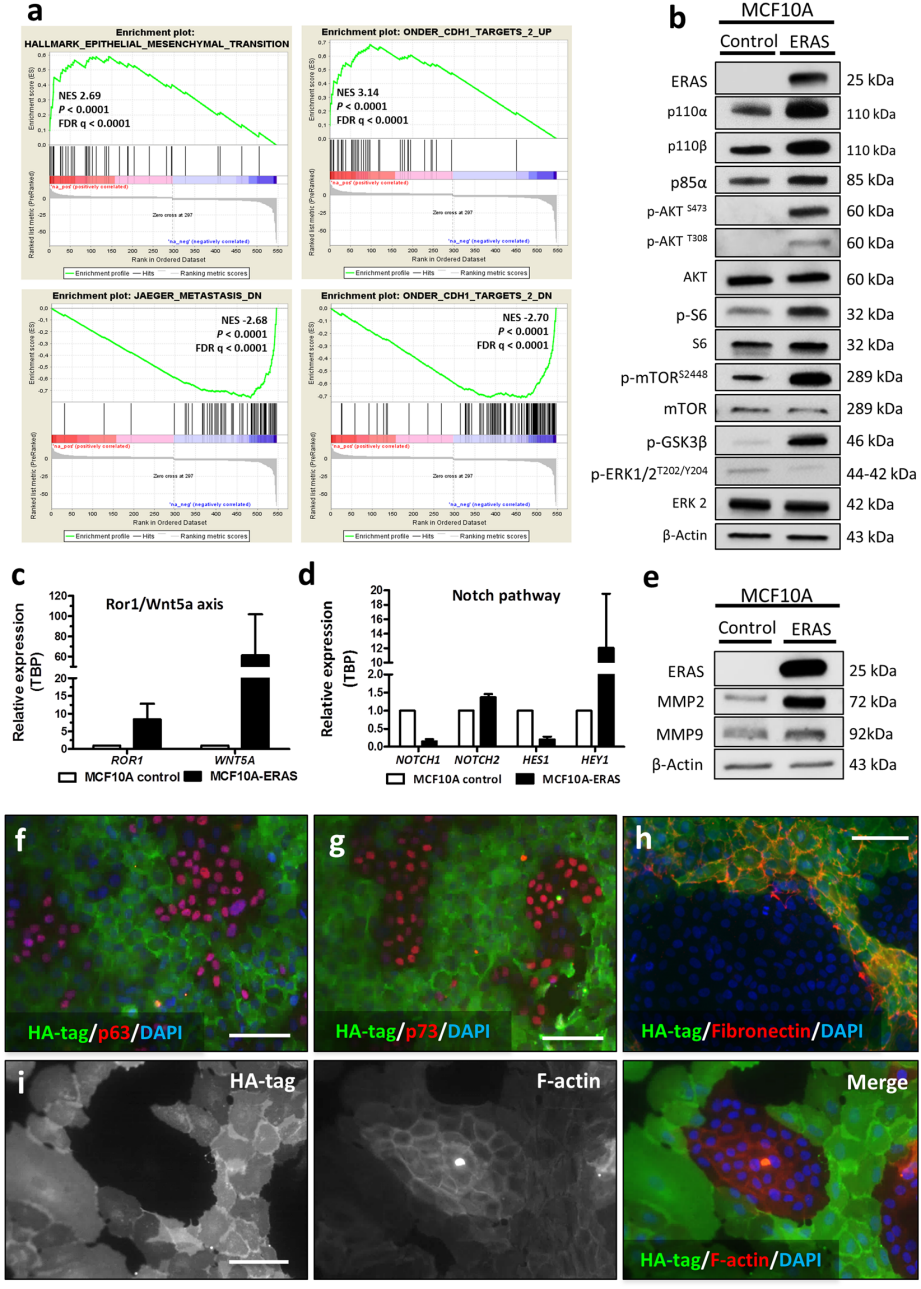
*ERAS* expression in MCF10A cells results in the alteration of multiple signaling pathways. (**a**) Examples of GSEA analysis of ranked genes up- or down-regulated by ERAS expression. In all cases, p-value and FDR were <0.0001. (**b**) Activation of proteins related to the PI3K/AKT/mTOR pathway in mammary gland cells expressing ERAS analyzed by western blot. (**c**,**d**) Gene-expression levels of (**c**) *ROR1 and WNT5A;* and (**d**) members of Notch pathway (*NOTCH1*, *NOTCH2*, *HES1* and *HEY1*) measured by RT-qPCR. (**e**) Western blot analysis showing an increase of MMP2 and MMP9 in MCF10A cells expressing ERAS. (**f**–**h**) Immunofluorescences of HA-tag (ERAS) and p53 family members p63 (**f**) p73 (**g**) and fibronectin (**h**) in co-cultures of MCF10A control and MCF10A-ERAS cells. Bar, 100 μm. (**i**) Immunofluorescences of HA-tag and F-actin (using Phalloidin-Atto 590) in co-cultures of MCF10A control and ERAS cells. Bar, 100 μm. In (**c** and **d**) relative values represent the means ± SD from three different experiments and were normalized with *TBP* gene. Uncropped blots for b and e are presented in Supplementary Fig. S6.

In order to understand the functionality of *ERAS* in mammary gland cells, we performed a transcriptome analysis of control and MCF10A-ERAS cells (Supplementary Table SIII). Gene ontology analysis of the differentially expressed genes revealed significant induction of genes involved in the EMT process, angiogenesis, cell migration, extracellular matrix organization and mammary neoplasms, among other functions. Most of the repressed genes could be assigned to downregulation of EMT, epithelial development and cell adhesion (Supplementary Fig. S2 and Supplementary Tables SIV and V). As a whole, these results indicate a strong shift of ERAS-expressing cells towards a malignant, invasive phenotype. Enrichment analysis using Gene Set Enrichment Analysis (GSEA) confirmed these results, finding that the upregulated genes were significant enriched in EMT genesets and the downregulated genes were enriched in genes repressed in metastasis (Fig. 5a). In addition, both the up- and down-regulated subsets of genes were strongly enriched with signatures for genes regulated after E-cadherin knockdown by RNAi in immortalized non-transformed mammary epithelium cells (Fig. 5a).

Although MAPK and PI3K pathways are the main effectors of Ras-GTPases, it has been shown in different cell types that ERAS cannot activate the MAPK pathway, instead signaling through PI3K to activate AKT^15,16,35–38^. To elucidate ERAS signal transduction mechanisms in MCF10A cells, we studied the expression of PI3K subunits (p110α, p110β and p85α), AKT and other downstream effector molecules (such as mTOR, S6 ribosomal protein or GSK3β). We confirmed that the PI3K/AKT/mTOR pathway was highly activated, while the MAPK pathway (measured as p-ERK) was not activated (Fig. 5b). In addition, our array results also revealed ERAS-induced alterations in other signaling pathways, that we validated using western blot, RT-qPCR and Immunofluorescence. Some of these alterations were upregulation of the ROR1/WNT5A axis (Fig. 5c), dysregulation of NOTCH signaling (Fig. 5d) and downregulation of p63 and p73 (Fig. 5f,g). We also observed an increase of metalloproteases (such as MMP2 and MMP9) and an enhancement of fibronectin, highlighting the possible implication of *ERAS* in the modification of the extracellular matrix (Fig. 5e,h). In addition, a decrease of F-actin fibers was observed, suggesting that ERAS expression leads to actin depolymerization (Fig. 5i). Taken together, our results support that *ERAS* is able to act through multiple pathways to promote growth, invasion and EMT.

### *ERAS* modulates the EMT process through downregulation of the miR-200 family

**Figure 6:**
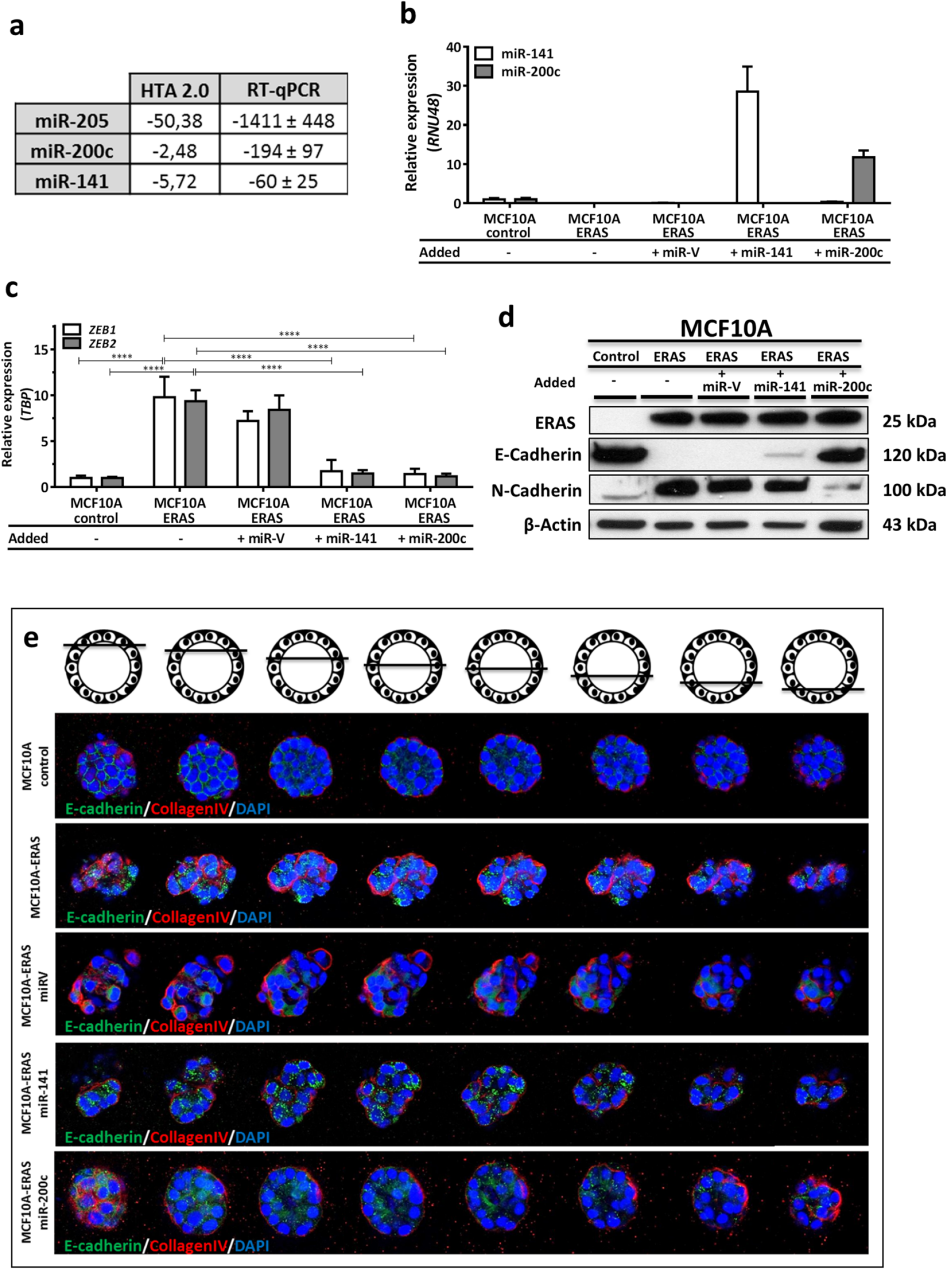
Downregulation of miR-200 family induces EMT in MCF10A-ERAS cells. (**a**) Fold changes of miR-205, miR-200c and miR-141 in MCF10A-ERAS cells with respect to control cells. Results from HTA 2.0 arrays (samples per duplicate) and quantitative RT-qPCR of three different experiments. (**b**) Expression levels of miR-141 and miR-200c after overexpression in MCF10A-ERAS cells. Relative values represent the means ± SD from three different experiments and were normalized with RNU48. (**c**) Gene-expression levels of transcriptional factors *ZEB1* and *ZEB2* in miR-141 and miR-200c- transfected MCF10A-ERAS cells, measured by RT-qPCR. Relative values represent the means ± SD from three different experiments and were normalized with *TBP* expression. (**d**) Western blot showing expression levels of E- and N-cadherin in MCF10A-ERAS cells overexpressing miR-141 and miR-200c. Uncropped blots are presented in Supplementary Fig. S6. (**e**) Recovery of the morphology of mammary acini in three-dimensional culture of ERAS-expressing MCF10A cells when overexpressing miR-141 and miR-200c. Images were taken by confocal microscopy. E-cadherin and collagen-IV were used to visualize cell junctions and basement membrane, respectively. Control and MCF10A-ERAS images are the same as in Fig. 2f.

Transcriptome analysis also revealed a sharp downregulation of some microRNAs (miRNAs) in MCF10A-ERAS with respect to control cells, specially miR-205, miR-200c and miR-141 (supplementary Table SIII). We confirmed the decrease of these miRNAs by quantitative RT-qPCR (Fig. 6a). miR-205 and the miR-200 family are frequently downregulated in several types of cancer. In breast cancer, miR-205 is considered a tumor suppressor, inhibiting cell growth, anchorage independent growth and invasion^39^. miR-200c and miR-141 have been implicated in the EMT process, targeting the transcription factors *ZEB1* and *ZEB2*^40,41^. In order to confirm if ERAS modulates the EMT process by downregulation of miR-200c and/or miR-141, we separately reintroduced both miRNAs in MCF10A-ERAS cells (Fig. 6b). Reintroduction of miR-200c in MCF10A-ERAS cells resulted in evident phenotypic changes, recovering a polygonal shape instead of the fusiform morphology of MCF10A-ERAS cells, and regaining adhesion to substrate (data not shown). Upon miR-200c overexpression, ERAS-induced increase of the transcription factors *ZEB1* and *ZEB2* was reverted, returning to levels similar to control cells (Fig. 6c). Furthermore, reintroduction of miR-200c increased levels of E-Cadherin to values similar to MCF10A control cells and decreased N-Cadherin expression. On the contrary, when miR-141 was overexpressed in MCF10A-ERAS cells, E-cadherin expression was only partially restored and N-Cadherin expression level did not change (Fig. 6d), even if levels of ZEB1 and ZEB2 returned to normal values, suggesting that miR-200c is the molecule that mediates the EMT process induced by ERAS. In addition, acini formed from MCF10A-ERAS-miR-200c cells recovered their spherical and hollow morphology in 3D cultures, while this recovery was partial in MCF10A-ERAS-miR-141 cells (Fig. 6e). Taken together, these results indicate that *ERAS* induces a strong downregulation of the cluster II of the miR-200 family, thereby causing a significant modulation of the EMT process, which seems to be mediated mainly by mir200c.

### *ERAS* increases growth rate and invasion, and decreases differentiation in xenograft tumors

**Figure 7:**
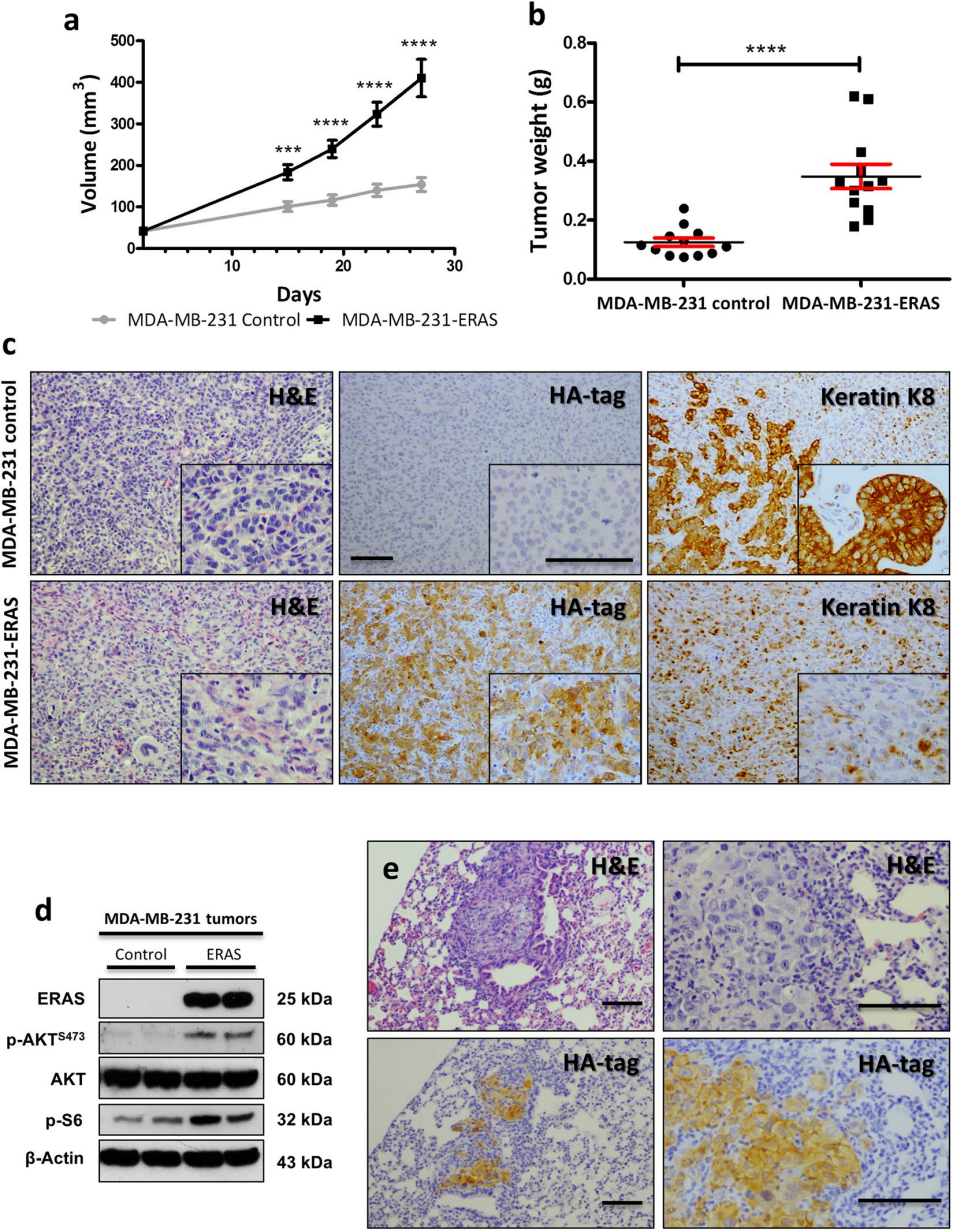
*ERAS* promotes the development of larger and more undifferentiated tumors when expressed in breast cancer cells. (**a**) Growth curves of tumors generated from MDA-MB-231 control and MDA-MB-231-ERAS cells in xenograft assays in nude mice. Tumors (n = 12 for each genotype) were measured on days 15, 19, 23 and 27 after injection. Tumors generated from ERAS-expressing cells grew faster than those arisen from control cells. (**b**) Weight of tumors extracted on day 27 after injection. (**c**) Histological characterization of xenograft tumors. Hematoxylin and eosin staining, and HA-tag and keratin K8 immunostaining are shown. Tumors-expressing ERAS did not show any foci of acinar differentiation expressing keratin K8. (**d**) Activation of AKT pathway in tumors generated from MDA-MB-231 analyzed by western blot. Uncropped blots are presented in Supplementary Fig. S6. (**e**) Histological images of lung metastasis of different mice injected with MDA-MB-231-ERAS cells. Hematoxylin and eosin staining and HA-tag immunostaining are shown. Asterisks show significant differences (***p < 0.001 and ****p < 0.0001). Bar, 100 μm. Data represent the means ± SEM.

To determine the role of ERAS in a tumoral context, we used the MDA-MB-231 cell line, derived from a metastatic breast adenocarcinoma. These cells form poorly differentiated adenocarcinomas when injected into nude mice. We generated MDA-MB-231 cells permanently expressing ERAS and analyzed the expression, localization and functionality of ERAS in these cells, which reproduced many of the changes seen in MCF10A cells (Supplementary Fig. S3a–c). When injected orthotopically into immunodeficient nude female mice, cells expressing ERAS formed tumors which grew earlier and larger than those from control cells (Fig. 7a,b). Tumors arising from MDA-MB-231-ERAS cells were histologically less differentiated, rarely showing acinar or ductal differentiation. We studied the expression of keratin K8, a luminal cell marker that is expressed in differentiated ductal breast carcinomas^42^. As expected, all control tumors presented differentiated foci positive to keratin K8, but these foci were not observed in any tumor expressing ERAS. Only scattered cells unable to form acinar or ductal structures remained positive to K8 staining, confirming the less differentiated status of tumors derived from MDA-MB-231-ERAS cells (Fig. 7c). These ERAS-expressing tumors also showed activation of the PI3K/AKT/mTOR pathway (Fig. 7d).

In order to assess whether *ERAS* enhanced the metastatic capacity of MDA-MB-231 cells, we surgically removed the primary tumors 27 days after the injection, and one month later we performed a histological study of three target tissues (lungs, liver and brain). Mice which had been injected with control cells did not develop metastasis in any tissue. On the contrary, we observed lung metastasis in fifty percent of the mice bearing MDA-MB-231-ERAS tumors (Supplementary Fig. S3d); these metastasis conserved ERAS expression (Fig. 7e,d). Thus, *ERAS* promotes tumor progression, invasion and dedifferentiation of MDA-MB-231 cells and increases the metastatic capacity of this cell line.

### *ERAS* is expressed in some human breast tumors

Expression of *ERAS* has been previously reported in human gastric cancer^35^. We analyzed *ERAS* expression in human breast tumors using data from The Cancer Genome Atlas (TCGA) breast cancer dataset^43,44^. Analysis of normal and tumoral breast samples from this dataset showed that approximately 8% of breast tumors express *ERAS*, although at low levels (Supplementary Fig. S4).

**Figure 8:**
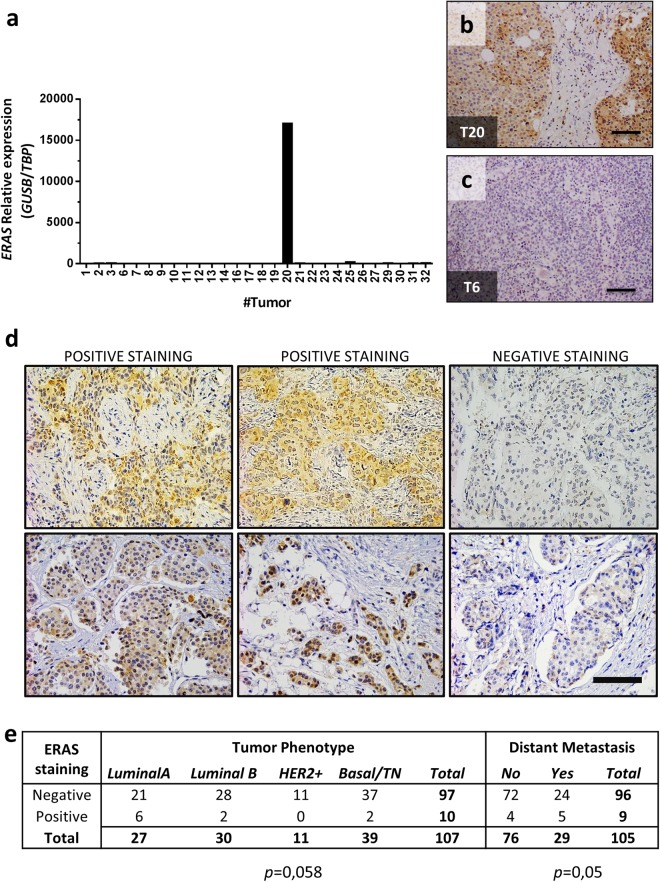
*ERAS* is expressed in human breast cancer. (**a**) Gene-expression levels of *ERAS* measured by RT-qPCR in a collection of breast tumors (n = 32). Expression values were normalized using *GUSB* and *TBP* genes. (**b**,**c**) Immunohistochemical staining of the ERAS-expressing (**b**) and a non-expressing (**c**) breast tumors using the ERAS D5G5J antibody. Bar, 100 μm. Tumor identifications (T6 and T20) are indicated in the lower left corner and correspond with numbers in A. (**d**) Immunohistochemical staining of ERAS in the IDC breast carcinoma samples. Examples from two different slides (upper and lower row) are shown. For each row, left and center images are positive for ERAS staining and right image is negative. Bar, 100 μm. (**e**) Statistical analysis showing correlation (Chi-square test) between ERAS staining, tumor phenotype and distant metastasis. Only samples with tumor phenotype and clinical parameters were included in the analysis. Significance is shown below.

To validate these results, we analyzed ERAS expression in a fully-characterized collection of 32 human breast tumors^9^. RT-qPCR analysis showed that one tumor (T20) presented high levels of *ERAS* mRNA, while the rest of the tumors produced null or background signals (Fig. 8a). Immunohistochemistry confirmed that tumor T20, a high-grade (G3), luminal B/Her2 type tumor was positive for ERAS antibody staining whereas all other tumors were negative (Fig. 8b,c). These results confirm that ERAS activation, although not frequent, can be found in breast tumors. We then addressed the biological significance of *ERAS* in an additional human breast cancer series, a cohort of formalin‐embedded grade 3 infiltrative ductal breast carcinomas (see^45^ for clinical and molecular description of the tumor series). This study revealed ERAS expression in 10 out of 107 tumors (9.3%, Fig. 8d). Six of these positive tumors were classified with a luminal A phenotype, two were luminal B and two were basal-like, which suggests that carcinomas with overexpression of ERAS mainly are luminal tumors. Although ERAS expression was not clearly correlated with the molecular phenotype of breast cancer (p = 0.058, Fig. 8e), its over-expression was significantly associated with distant metastasis (p = 0.05, Fig. 8e) and reduced metastasis-free survival (p = 0.047, Supplementary Fig. S5) suggesting that ERAS expression could be considered as a poor prognosis biomarker in breast cancer.

## Discussion

Generation of mammary tumors using the SB transposon system has allowed us to identify *Eras* as a gene involved in the development and progression of murine mammary tumors. The SB mutagenic transposon has been widely used for identification of tumor suppressor genes and oncogenes in different transgenic mouse models^7^. Although seldom studied, insertions in *Eras* have been reported in other types of murine tumors generated by the SB system, including melanoma and non-melanoma skin cancers^17,38^, leukemia/lymphoma^46^, osteosarcoma^47^ and malignant peripheral nerve sheath tumors^48^. In all cases, transposons were inserted 5′ upstream of the *Eras* gene and in the same transcriptional direction, resulting in *Eras* activation. Moreover, using retroviral insertional mutagenesis *Eras* was identified as a possible oncogene in murine lymphomas^49^ and mammary gland tumors^50^. Altogether, these data underpin the ability of *Eras* to act as an oncogene in multiple tissues.

The human *ERAS* gene was considered a pseudogene for a long time and probes for *ERAS* in gene-expression arrays have only recently been included (for instance, widely used whole human genome expression arrays such as Affymetrix U133 did not include probes for ERAS). Therefore, expression of this gene has been poorly characterized, and its role in development and tumorigenesis is still largely unknown. Nonetheless, several studies have shown that *ERAS* is aberrantly expressed in human gastric cancer^35,51^, and a recent report has found evidence of a highly oncogenic *USP9X-ERAS* fusion in colorectal cancer^52^. Here, we describe the expression of *ERAS* in a small subset of breast tumors. In a collection of 32 human breast tumors, we found unequivocal expression of ERAS at the mRNA and protein levels. In addition, the analysis of *ERAS* expression data from TCGA also suggests that a low percentage of breast tumors express *ERAS*, and immunohistochemical analysis of a grade 3 breast tumor tissue microarray confirmed that ERAS is aberrantly expressed in less than 10% of tumors. Nonetheless, our data suggest that *ERAS* expression tends to occur in tumors with luminal phenotype and distant metastases, supporting the idea of ERAS having a role in breast tumor malignancy.

Results from our forced expression experiments indicate that inappropriate expression of ERAS in human non-malignant breast MCF10A cells is sufficient for causing all processes that define EMT: increase in motility, loss of adhesion, reorganization of the cytoskeleton, cadherin switch, induction of EMT genes and transcription factors^22^. In addition, we also show that ERAS increases tumor growth and metastasis in MBA-MD-231 cells. In MCF10A cells, we have seen that ERAS downregulates miR-205, miR-141 and miR-200c; these are tumor suppressor miRNAs whose reduced expression has been consistently associated to cell growth, invasion, migration, cancer stem cells, EMT and breast cancer^39–41,53,54^. Importantly, we show here that miR-200c plays a principal role modulating the EMT process induced by ERAS expression, since this process can be reversed by miR-200c overexpression. miR-200c has been shown to have therapeutic effects in an *in vivo* model of breast cancer, reducing dedifferentiation and tumor proliferation^54^, and its expression has been associated to significantly better overall survival in human patients^55^. In this respect, metformin has been shown to induce the expression of miR-200c^56^, suggesting a way for the specific therapeutic treatment of ERAS-expressing breast tumors.

It has been reported that *ERAS* may provide resistance to chemotherapy in different human tumorigenic cells, such as neuroblastoma^37^, gastric cancer^57^ and melanoma^38^, by activation of the PI3K/AKT/mTOR pathway, promoting both DNA replication and transcription. It is interesting to note that ERAS-mediated resistance can be reverted by inhibitors of PI3K^37^, AKT^38^, mTOR and topoisomerase-1^57^. Here, we have confirmed that ERAS acts through the PI3K/AKT pathway in breast cells. Moreover, we show that expression of *ERAS* results in the activation or downregulation of multiple genes and signaling pathways, providing several possible targets for treatment of ERAS-expressing tumors. For instance, we have observed an upregulation of the receptor ROR1, which, strikingly, is expressed during normal embryonic and fetal development but absent in most mature tissues, and is also overexpressed in aggressive breast tumors^58,59^.

Since ERAS is a Ras GTPase that is constitutively active and does not require mutation events, it follows that it must be tightly inactivated to restrain its strong tumorigenic properties. The mechanism by which the *ERAS* gene is activated in tumorigenic cells is still not clear. In one case, activation of *ERAS* by gene fusion with an active promoter has been described^52^. But in addition, several studies suggest that *ERAS* silencing is associated with epigenetic transcriptional regulation, by mechanisms that include methylation of CpG islands^48,60,61^ and histone deacetylation^62^. In fact, preliminary studies from our laboratory (not shown) also suggest that *ERAS* activation in breast tumors could be mediated by epigenetic processes. These epigenetic modifications are not unexpected: *ERAS* is localized in the X chromosome, and abnormalities in this chromosome are common in breast tumors. The inactive X chromosome (Xi) is epigenetically unstable and this frequently leads to alterations in transcription in breast cancer. Inactivation of the Xi chromosome is frequently reverted in breast tumors, having been observed an association with the overexpression of X-linked genes^63,64^. Therefore, epigenetic instability of the Xi chromosome could lead to *ERAS* reactivation.

As a conclusion, in this report we show that ERAS is expressed in a subset of breast tumors and demonstrate that ERAS expression in human normal mammary gland cells leads to a strong induction of EMT and to the development of tumoral and stem-cell features, which can be at least in part reverted by reactivation of miR-200c. These results are clinically relevant, since they open up new ways for therapeutic intervention.

## Methods

### Human tumors

Patient samples used for immunohistochemistry and qRT-PCR were provided by the Biobanco i + 12 in the Hospital 12 de Octubre integrated in the Spanish Hospital Biobanks Network (RetBioH; www.redbiobancos.es) following standard operation procedures with appropriate approval of the Ethical and Scientific Committees (Reference: CEIC 15/094). Tumor data for these samples are presented in Supplementary Table SVI.

Tissue microarrays included a total of 107 infiltrating ductal breast carcinomas (IDC) acquired from the archives of the Pathology Department of MD Anderson Cancer Center Madrid, Madrid, Spain. Description and clinical data of these tumor samples have been provided^45^, and additional information is provided in Supplementary Table SVII. Patients underwent surgery between 2003 and 2004. The mean patient age at surgery was 59.2 years (range, 40 to 79 years). All tumors were grade 3. Histological and immunohistochemical studies were all carried out on formalin-fixed, paraffin-embedded tissue samples. This study was performed following standard ethical procedures of the Spanish regulation (Ley de Investigación Orgánica Biomédica, 14 July 2007) and was approved by the ethic committees of the La Paz Hospital, and MD Anderson Cancer Center Madrid, Madrid, Spain. ERAS staining results were independently evaluated by three authors (C.S.-C., A.R. and M.N.) and classified as positive or negative.

### Animals

Double K5-SB11/T2Onc2 (named as SB/T2) transgenic mice, containing both the SB11 and T2Onc2 transgenes were generated by interbreeding of hemizygous SB and T2 mice, as described^17^. Heterozygous p53+/− transgenic mice were obtained by mating conditional mutants Trp53F2-10 mice^65^, that carried floxed Trp53 alleles to K5-Cre transgenic female mice^66^. Triple transgenic K5-SB11/T2Onc2/p53+/− mice (SB/T2/p53+/−) were generated by mating hemizygous SB/T2 to heterozygous p53+/− mice. Mice were in a mixed (FVB, C57BL6J and DBA2J) genetic background. Animals were typed by PCR. Transposon integration analysis has already been described^9^. All procedures involving mice were approved by the Institutional Organism for Animal Welfare (OEBA) and according to the European, Spanish and local regulations.

### Cell culture, DNA constructs, transfection and lentiviral production

MCF10A and MDA-MB-231 cells were purchased from American Type Culture Collection (ATCC). Monolayer and three-dimensional culture of MCF10A cells was performed as described^20^. MCF10A-ERAS cells were grown in plates coated with 0.1% gelatin due to their limited adhesion. MCF10A control cells were also grown in gelatin-coated plates to avoid substrate effect. MDA-MB-231 cells were cultured in Dulbecco’s Modified Eagle Medium (DMEM) with Glutamax and high glucose (Gibco) supplemented with 10% of fetal bovine serum (FBS) and non-essential amino acids (Gibco). All cell lines were routinely tested for mycoplasma contamination.

The *ERAS* gene was amplified from human genomic DNA using both forward (5′-ACAAGCTTAATGTACCCATACGATGTTCCAGATTACGCTGAGCTGCCAACAAAGCCTGGC-3′) and reverse (5′-CTCGCCGGCGTTCAGGCCACAGAGCAGCCACAGTG-3′) primers and was cloned into pRc/CAG vector^18^, which confers resistance to G418. The HA epitope (YPYDVPDYA) was inserted in frame in the 5′-end of the sequence. Control and HA-hERAS plasmids were transfected into MCF10A and MDA-MB-231 cell lines by electroporation using a SE CellLine 4D-Nucleofector X-Kit (Lonza) according to the manufacturer’s instructions. Pools of more than one hundred clones of MCF10A and MDA-MB-231 cells were selected with G418 (300 µg/ml and 1 mg/ml, respectively).

Third generation lentiviral vectors encoding miR-200c and miR-141 precursors^67^ and empty vector miR-V were co-transfected with packaging plasmids (pMDLg/pRRE, pRSV-REV and pMD2.G) in 293T cells using Polyethyleneimine (Polysciences, Inc). 48 hour-supernatants were collected, passed through a 0.45 µm filter and ultra-centrifuged to concentrate lentiviruses. Cells were infected and GFP-positive cells were selected by flow cytometry.

### RNA isolation and quantitative RT-qPCR

Total RNA from 80% confluence cultured cells was isolated using miRNeasy Mini Kit (Qiagen) and DNA was eliminated with an Rnase-Free DNase Set (Qiagen) according to the manufacturer’s instructions. Total RNA from human tumors was isolated from formalin-fixed, paraffin-embedded tissue sections using miRNeasy FFPE Kit (Qiagen). The reverse transcription reaction was performed using the High Capacity cDNA Reverse Transcription Kit (Applied Biosystems) for mRNA and TaqMan MicroRNA Reverse Transcription Kit (Applied Biosystems) for miRNA. Quantitative qRT-PCR was performed in a 7500 Fast Real Time PCR System using GoTaq qPCR Master Mix (Promega) for mRNA and TaqMan Universal PCR Master Mix (Applied Biosystems) for miRNA. The sequences of the oligonucleotides used are listed in Supplementary Table SVIII. For miRNA, TaqMan Probes were used. For normalization of gene expression of cells, *TBP* and *RNU48* were used as reference for mRNA and miRNA respectively. For human tissue, we used a combination of *TBP* and *GUSB* as endogenous control for normalization, as selected by NormFinder^68^ and geNorm^69^. Fold changes were calculated using the formula 2^(log2B−log2A)^, being A the expression level of control cells and B the expression level of ERAS cells.

### Immunohistochemistry and immunofluorescence

All antibodies used are listed in Supplementary Table SIX. For immunohistochemistry, tissues were fixed in buffered formalin and embedded in paraffin. Slides were deparaffinized and antigen retrieval was performed with citric acid buffer (pH 6) using a microwave oven (3 minutes at 900 W and 15 minutes at 150 W) for HA-tag and keratin K8 antibodies or with Dako Target Retrieval Solution (pH 9) using a pressure cooker for the D5G5J ERAS antibody. Endogenous peroxidase was inhibited with hydrogen peroxide (0.3%) in methanol. For immunofluorescence, cells were grown on coverslips, fixed with buffered paraformaldehyde (4%) and permeabilized with PBS containing 0.1% of Triton X-100. In all cases, unspecific epitopes were blocked with PBS containing 10% of horse serum and then primary antibodies were incubated overnight at 4 °C diluted in blocking solution. For immunohistochemistry, signal was amplified with biotin-avidin-peroxidase system (ABC elite kit Vector) and visualized using a DAB Kit (Vector Laboratories). In immunofluorescence, DAPI was used to stain nuclei and pictures were taken with a Zeiss Axioplan fluorescence microscope. All immunofluorescence analyses were performed at least three times.

Immunofluorescence staining of MCF10A acini cultured in matrigel was performed as described^20^. Mammospheres were fixed fifteen days after plating. Experiments were performed in duplicate in 4-well culture slides (Corning BioCoat). 50–100 mammospheres were visually inspected in each well for morphology and immunofluorescent labeling of targeted proteins. Mammosphere images were taken with a Zeiss LSM510 META ConfoCor 3 spectral confocal microscope.

### Immunoblot analysis

All antibodies used are listed in Supplementary Table S IX. Proteins extracts were isolated from cells or homogenized tissues in lysis buffer. Gel electrophoresis and proteins transfer (Bio-Rad) were performed according to manufacturer’s instructions. Membranes were blocked with 5% w/v nonfat dry milk in 0.1% Tween-20 PBS. Primary and secondary antibodies were diluted in blocking solution. Blots were visualized with Clarity Western ECL substrate (Bio-Rad) using a ChemiDoc MP System and ImageLab software (Bio-Rad). β-Actin was used as a loading control. All immunoblot analyses were performed at least three times.

### Proliferation and wound-healing assays

We used a colorimetric XTT cell proliferation assay kit (Roche) for quantification of cell proliferation. For these assays, 4,000 cells were plated in sextuplicate on 96-well plates per each day. The absorbance value (at 492 nm) of the first day after plating cells was taken as reference for normalization. Absorbance was measured using a microplate reader.

For migration assays, cells were grown in triplicate on 6-well dishes. Upon confluence, cells were treated with mitomycin C (Sigma Aldrich, 1 μg/ml) to inhibit proliferation and then two wounds were generated in each well using a pipette tip. Four images for each cell type were taken at the indicated time points and migration areas of the cells in the leading edge of the wound were measured using ImageJ software.

### Cell cycle and flow cytometry analysis

For cell cycle, cells were grown in triplicate on 6-well dishes in different confluence conditions. Next, cells were trypsinized, fixed with cold 70% ethanol and stored overnight at 4 °C. Cells were washed and incubated with PBS containing 0.05% of NP40 and 2 µg/ml of DAPI for 4 hours at 4 °C. To quantify cell surface markers (EpCAM, CD49f, CD44 and CD24), 1.5 × 10^5^ cells were plated in triplicate on 6-well dishes. When cells reached 70–80% confluence they were trypsinized, washed and incubated with primary antibodies (Supplementary Table SIX). Data were acquired on an LSR Fortessa flow cytometer and analyzed using FlowJo software.

### Orthotopic mammary xenografts assays

For mammary fat pad orthotopic xenograft experiments, 2.5 × 10^6^ MDA-MB-231 cells were injected bilaterally into the fourth mammary fat pad of 8-week female *nu/nu* mice (n = 6 for each group; control and ERAS cells). Cells were injected with matrigel (BD Biosciences) in a 1:1 ratio (final volume = 100 μl). Tumor size was measured at the indicated time points using a caliper, and tumor volume was calculated as 4π/3 × (length/2 × (width/2)^2^). Xenograft tumors were surgically removed 27 days after the injection. One month later, mice were sacrificed to assess the metastatic capacity of cells in lungs, liver and brain.

### Gene expression profiling and analysis

RNA from subconfluent cultured cells was extracted as described above. Gene Expression analysis was performed using HTA 2.0 arrays (Affymetrix). Data were normalized using Expression Profiler (Affymetrix) and differential expression was tested using TAC (Affymetrix). Gene Ontology analysis was performed using Toppgene^70^. GSEA^71^ was used to compare the gene expression pattern of MCF10A-ERAS cells with other signatures.

### TCGA and cBioPortal data analysis

Expression data of human breast tumors were obtained from the cBioPortal for Cancer Genomics (www.cbioportal.org; dataset Cell 2015)^43,44^; RNASeq data were downloaded from the Genomic Data Commons Data Portal (https://portal.gdc.cancer.gov/projects?filters=~%28op~%27and~content~%28~%28op~%27in~content~%28field~%27projects.primary_site~value~%28~%27Breast%29%29%29%29%29). Threshold for *ERAS* expression was defined as values higher than the mean value of control samples from the TCGA dataset plus twice the standard deviation. This equals to 0.08 reads per kilobase million (RPKM). Correlations were calculated by using the Pearson correlation coefficient. In all cases, p-values < 0.05 were considered significant and data are expressed as means ± SD.

### Statistical analysis

For proliferation, wound-healing and flow cytometry assays, p-values were determined by using the unpaired, two-tailed Student t test. All experiments were performed at least three times. P-values < 0.05 were considered significant and data are expressed as means ± SEM for proliferation assays and as means ± SD for wound-healing and flow cytometry assays.

To compare volume and weight from tumor xenografts, p-values were determined by using the unpaired, two-tailed Student t test. P-values < 0.05 were considered significant and data are expressed as means ± SEM.

The χ2 or Fisher’s exact tests were used to test associations between ERAS expression and pathological characteristic of breast IDC as categorical variables. All tests were two-tailed and 95% confidence intervals (CIs) were used. Values of p < 0.05 were considered statistically significant. Distant metastasis free survival was defined as the time from the date of diagnosis to the date of development of novel distant metastasis. Survival curves were generated using the Kaplan-Meier method. The p-values are based on log-rank test. Statistical analysis were performed in R and using the Survival package and SPSS Statistics 17.0.

### Ethical approval and informed consent

The use of human samples in the present study was approved by the Ethics Committee for Clinical Research (CEIC) of the 12 de Octubre University Hospital (Reference 15/094). For all human specimens, informed consent was obtained from all subjects in accordance with the requirements. All animal studies were approved by the Institutional Animal Care and Use Committee (OEBA) of CIEMAT and by the Ethical Committee of Comunidad de Madrid (PROEX 035/13). All methods used in both animal and human experiments were performed in accordance with the relevant guidelines and regulations.

## Electronic supplementary material

Supplementary Figures S1-S6

Supplementary Tables SI-SIX

## Data Availability

All transposon insertion sites corresponding to the three animals bearing insertions in *Eras* are shown in Supplementary Table S II. Microarray data that support the findings of this study have been deposited in the GEO repository with the accession code GSE104859. All the datasets supporting the conclusions of this article are included within the article and its additional files.
