# Supplementary material for: The Ras-related gene ERAS is involved in human and murine breast cancer
Source: Sci Rep. 2018 Aug 29;8:13038. doi: 10.1038/s41598-018-31326-4 (PMC6115423; doi:10.1038/s41598-018-31326-4)
Supplement: Supplementary file 1 — Supplementary Figures S1-S6 [file 41598_2018_31326_MOESM1_ESM.pdf]

Supplementary Figures S1-S6

The Ras-related gene *ERAS* is involved in human and murine breast cancer

Cristian Suárez-Cabrera, Bárbara de la Peña, Laura L. González, Angustias Page, Mónica Martínez-Fernández, M. Llanos Casanova, Jesús M. Paramio, Alejandro Rojo-Sebastián, Gema Moreno-Bueno, Alicia Maroto, Ángel Ramírez and Manuel Navarro

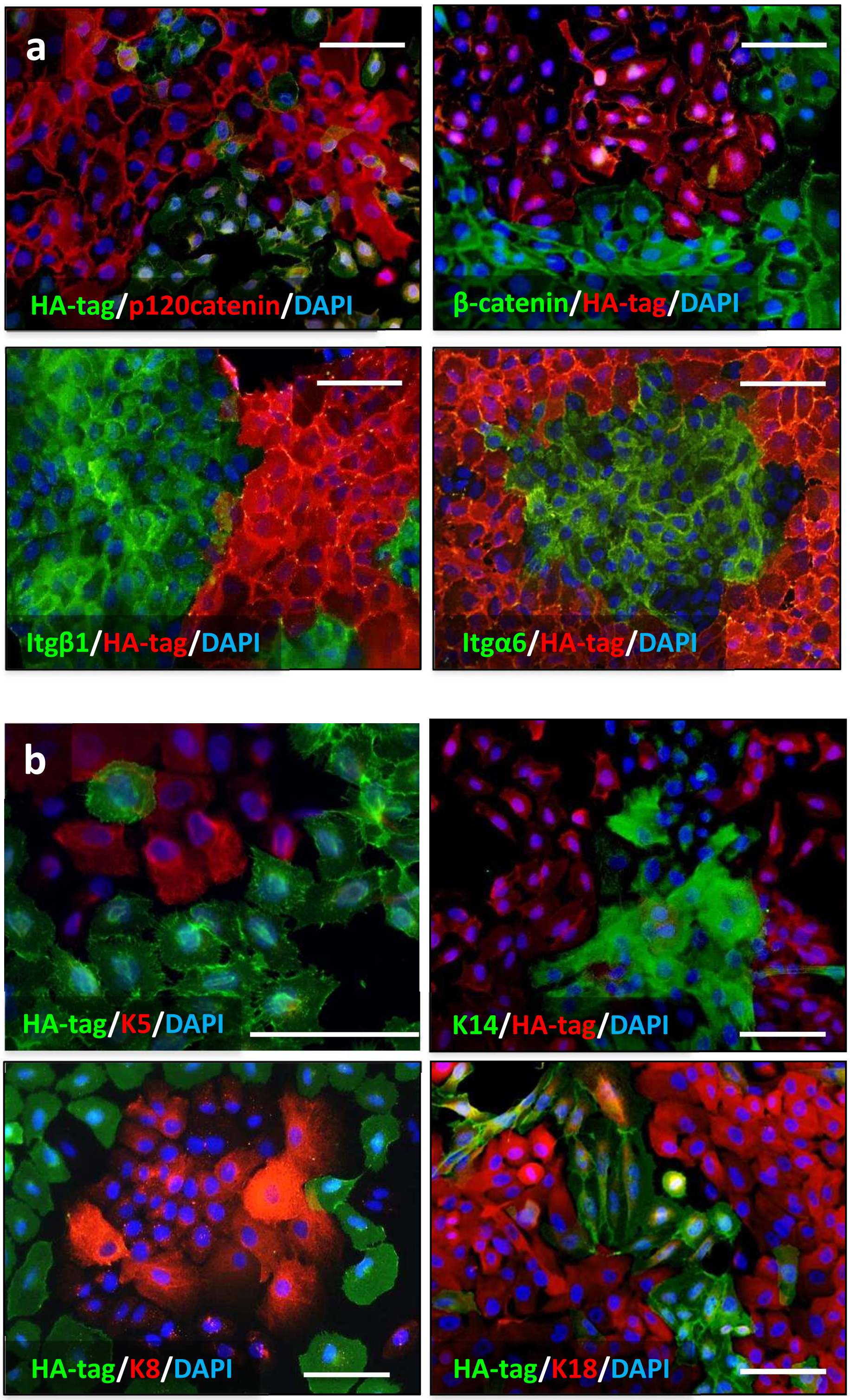

Supplementary Figure S1. Inhibition of adhesion molecules and keratins in MCF10A-ERAS cells. (a) Immunofluorescences of ERAS (HA-tag), p120-catenin, β-catenin, α6 and β1 integrins in co-cultures of MCF10A-control and MCF10A-ERAS cells. Bar, 100μm. (b) Immunofluorescences of ERAS (HA-tag), keratins K5, K14, K8 and K18 in co-cultures of MCF10A control and MCF10A-ERAS cells. Bar, 100μm.

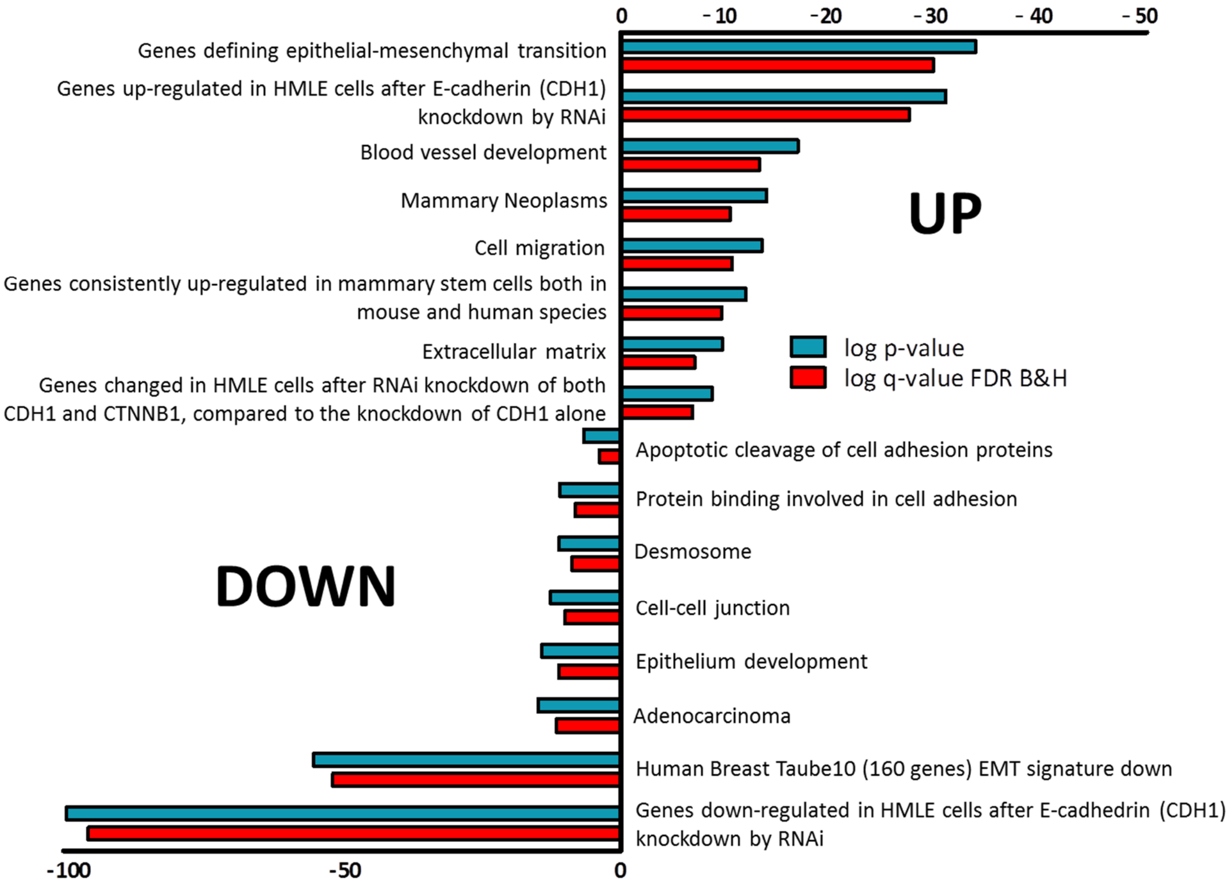

Supplementary Figure S2. Graph showing the log10 p-value and Benjamini and Hochberg´s corrected false discovery rate (FDR B&H) of selected functions from Gene Ontology analysis of genes up- or down-regulated by ERAS.

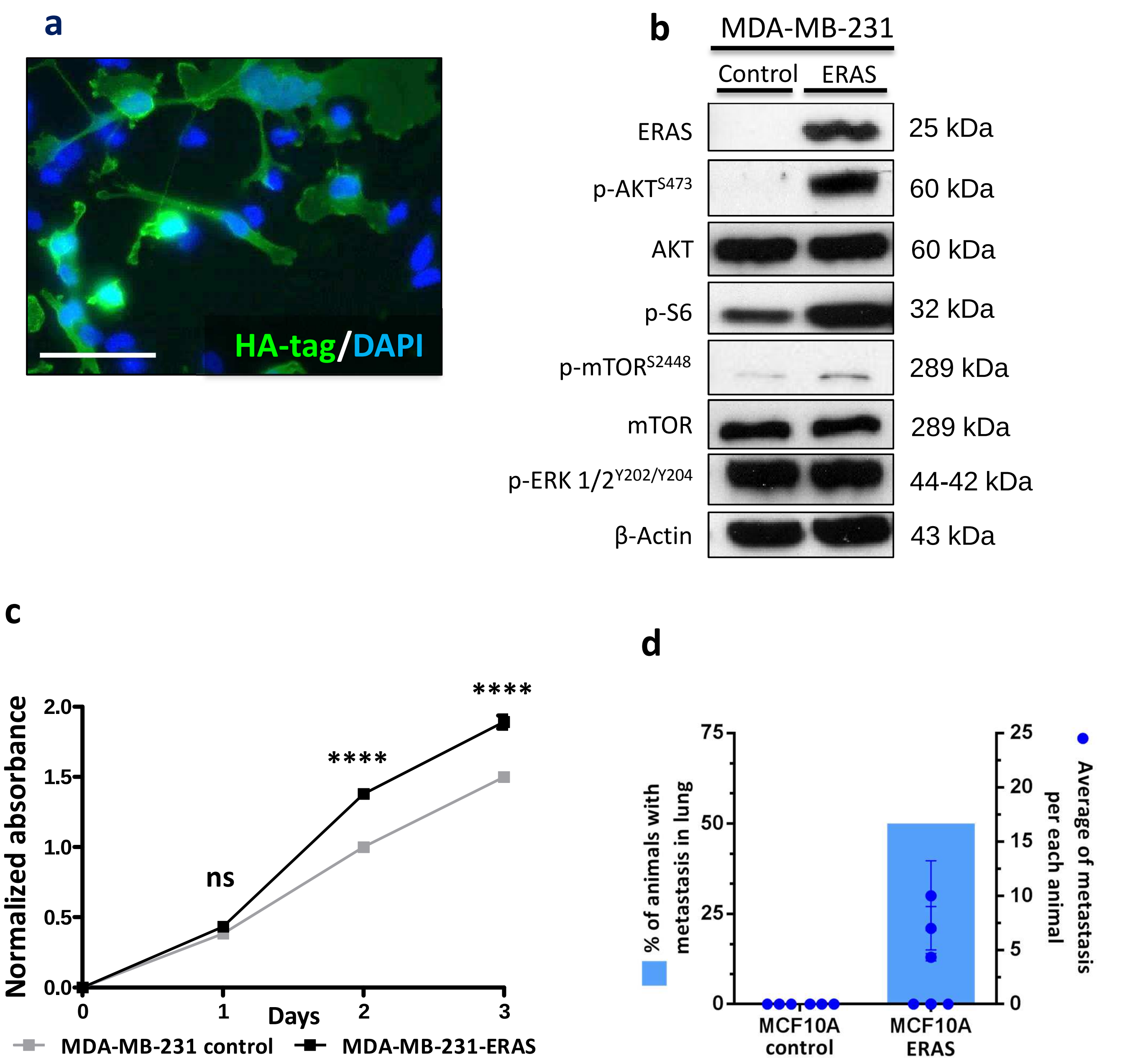

Supplementary Figure S3. ERAS expression, localization and functionality in MDA-MB-231 cells. (a) Immunofluorescence showing HA-tag staining in MDA-MB-231-ERAS transfected cells. Plasmatic membrane and cytoplasm were stained. Bar, 100µm (b) Activation of PI3K/AKT/mTOR pathway in MDA-MB-231-ERAS cells analyzed by western blot. Uncropped blots are presented in Supplementary Fig. S6. (c) Representative growth curves of MBA-MB-231 control and MBA-MB-231-ERAS cells. Proliferation was measured using XTT proliferation assay. Data represent the means ± SEM. (d) Histogram displaying the number of animals with metastasis in lung per genotype (left axis) and the average number of metastasis per animal (right axis). No tumors were detected in liver or brain.

a

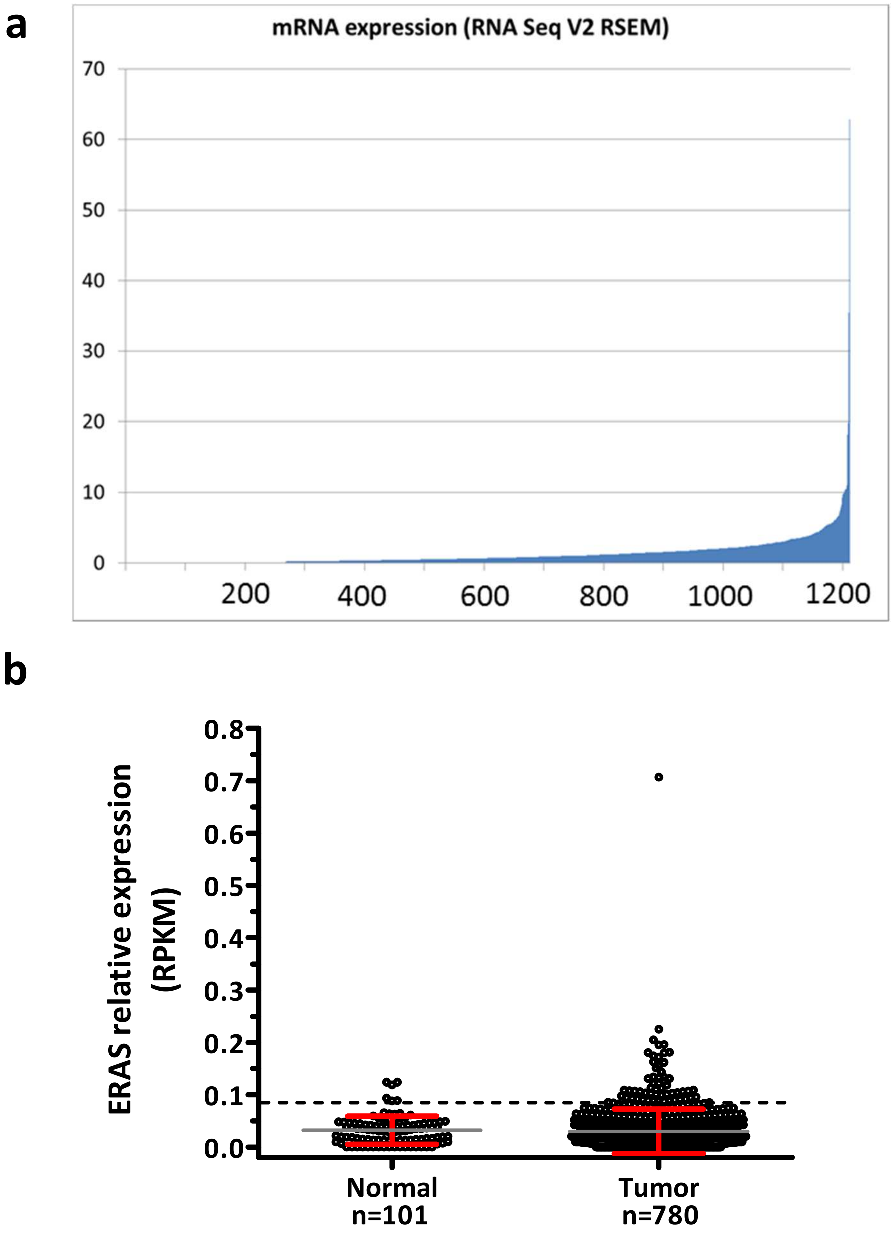

Supplementary Figure S4. *ERAS* is expressed in some human breast tumors. (a) Expression of the *ERAS* gene across 1200 TCGA RNAseq data sets from breast cancer samples. RNAseq V2 RSEM normalized datasets (x-axis) were ranked from low to high expression**.** (b) Expression levels (RPKM) of *ERAS* in normal and tumoral breast samples. The mean ± SD are shown. Dashed line represents the threshold value (healthy tissue mean + 2SD) for considering *ERAS* expression**.** Data were obtained from the Genomic Data Commons Data Portal (NIH) at https://portal.gdc.cancer.gov/projects?filters=~%28op~%27and~content~%28~%28op~%27in~content~%28field~%27projects.primary_site~value~%28~%27Breast%29%29%29%29%29.

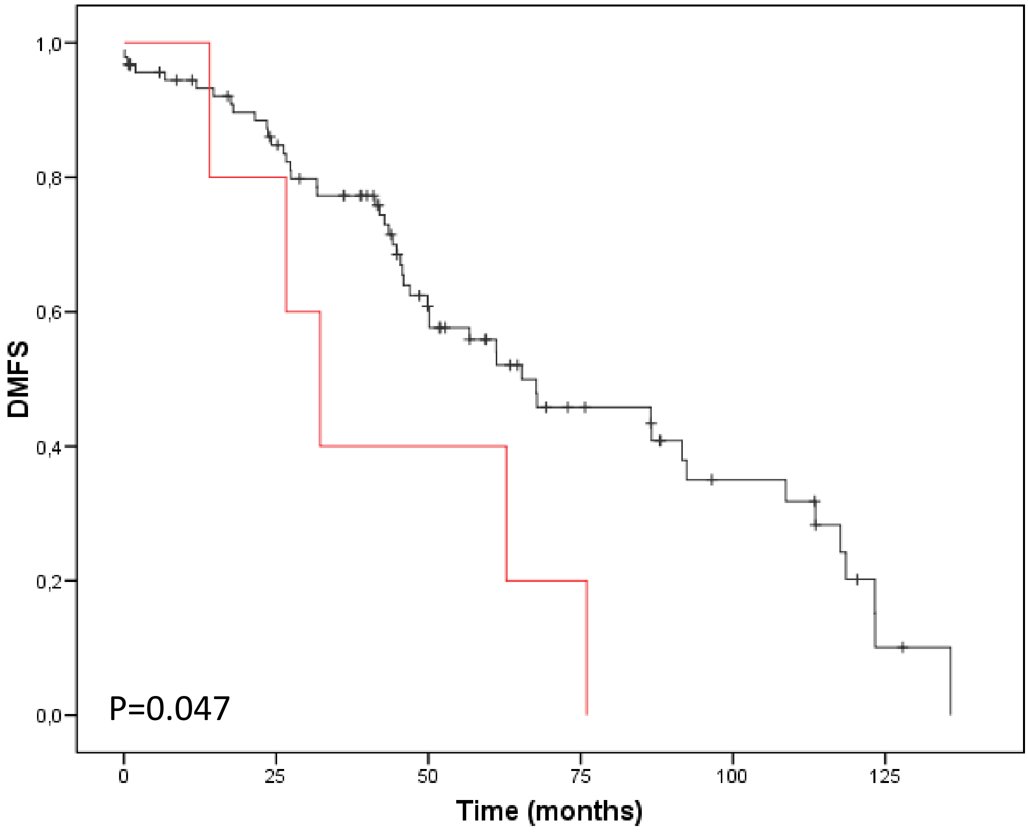

Supplementary Figure S5. ERAS expression associates with distant metastasis free survival. Distant metastasis free survival (DMFS) curves in breast IDC in relation to ERAS expression (p value 0,047) Statistical differences, *p-value*, were calculated via log-rank test. Red line: patients with ERAS expression; black line: patients without ERAS expression.

Original blots for Fig. 2b and 4d

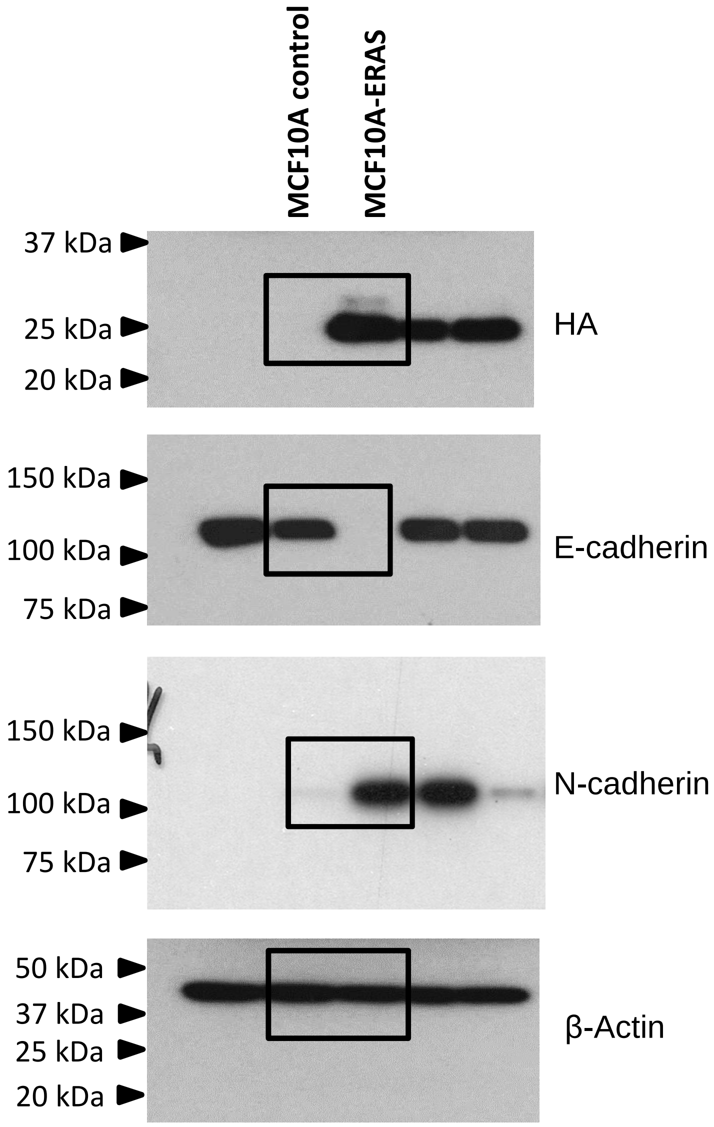

Original blots for Fig. 2c

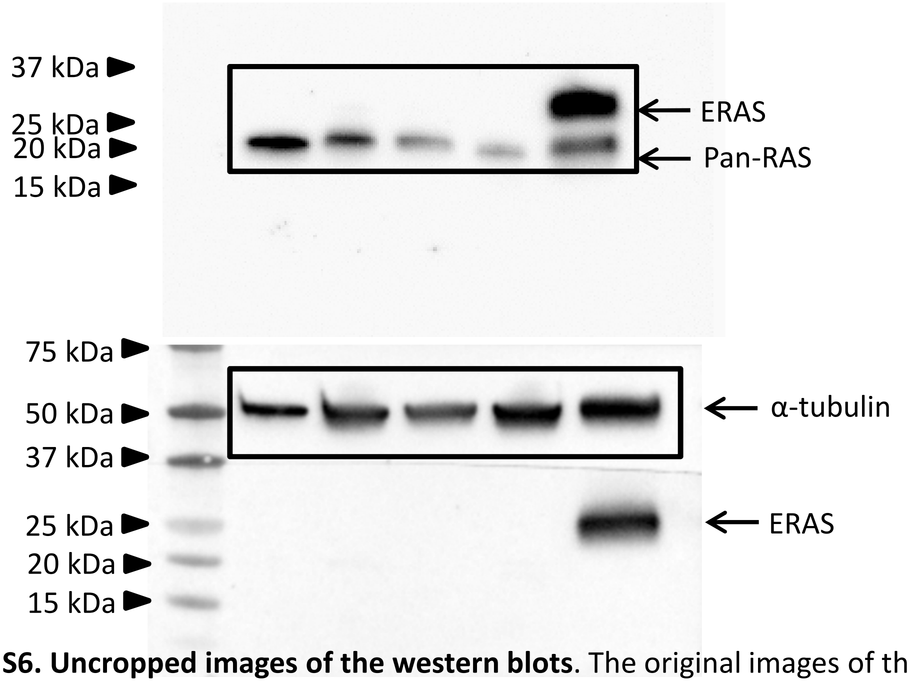

Supplementary Figure S6. Uncropped images of the western blots. The original images of the western blots shown in figures 2B, 2C, 4D, 5B, 5E, 6D and S3B are shown. The boxed sections indicate the regions displayed in the manuscript figures.

Original blots for Fig. 5b

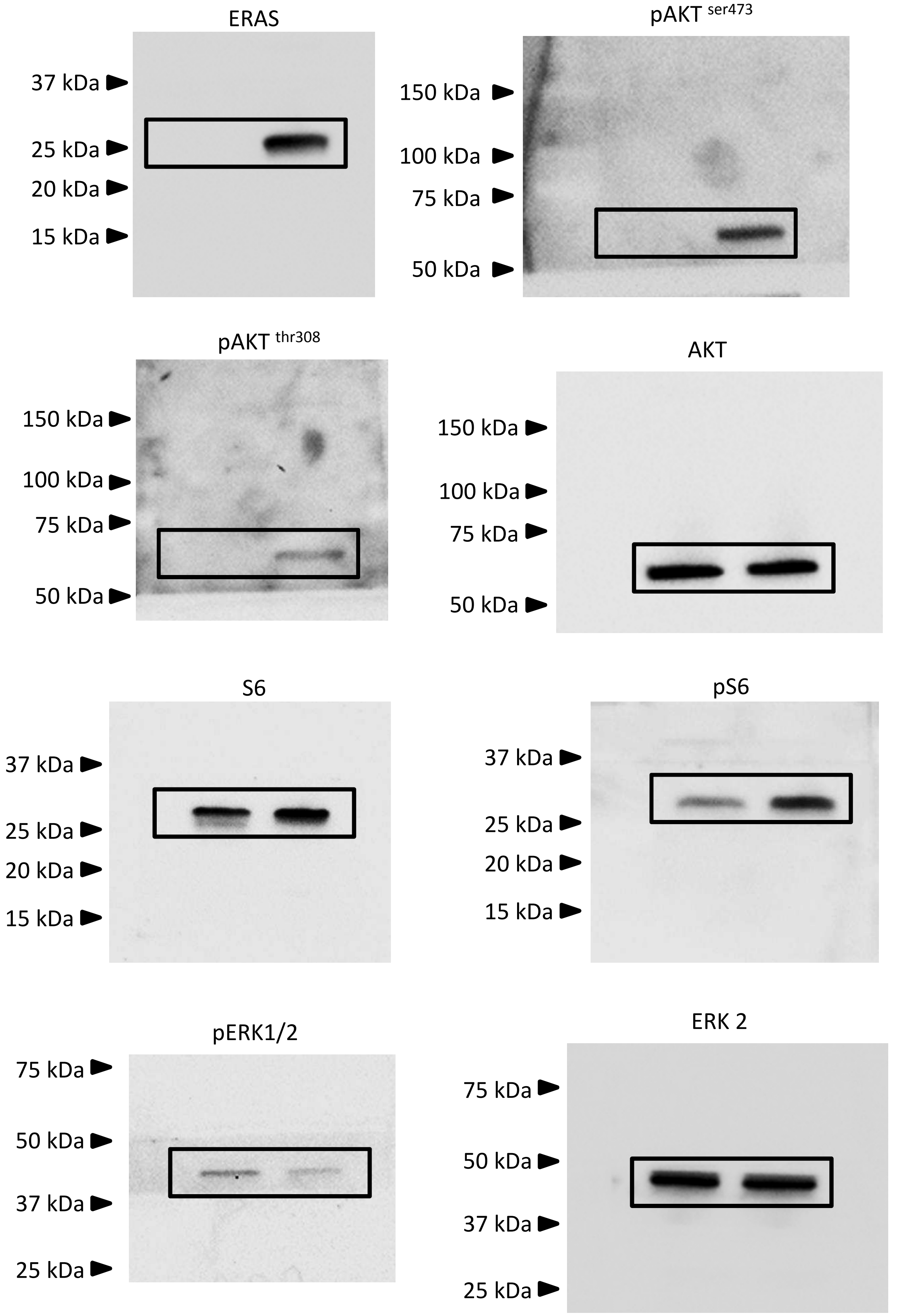

Supplementary Figure S6 (cont.)

Original blots for Fig. 5b (cont.)

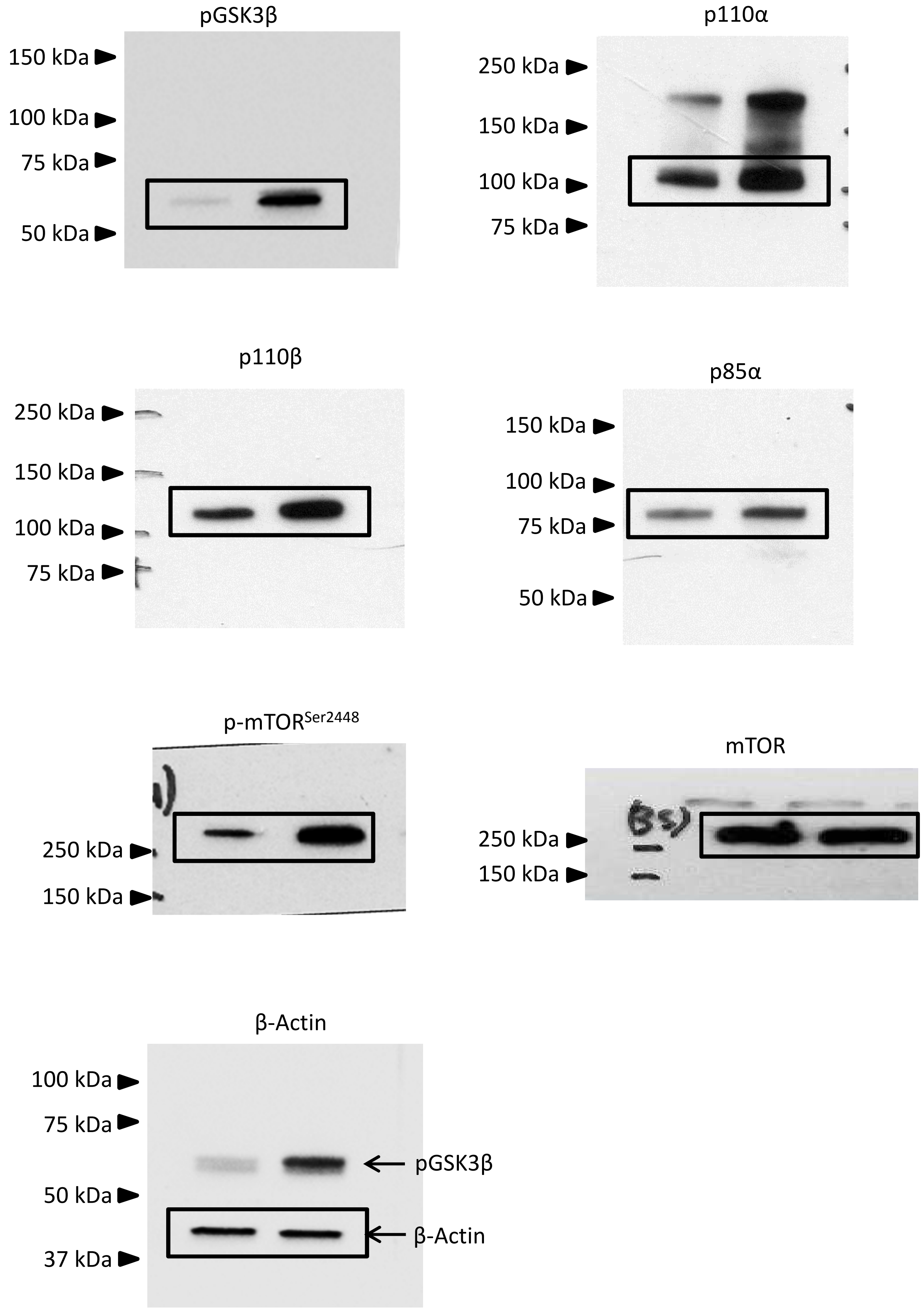

Supplementary Figure S6 (cont.)

Original blots for Fig. 5e

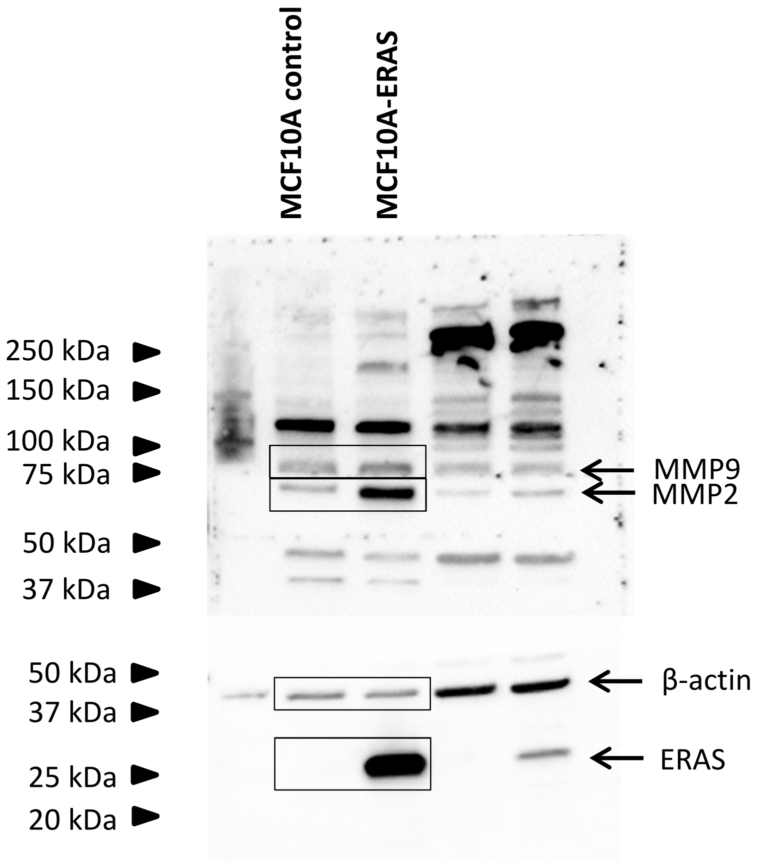

Original blots for Fig. 6d

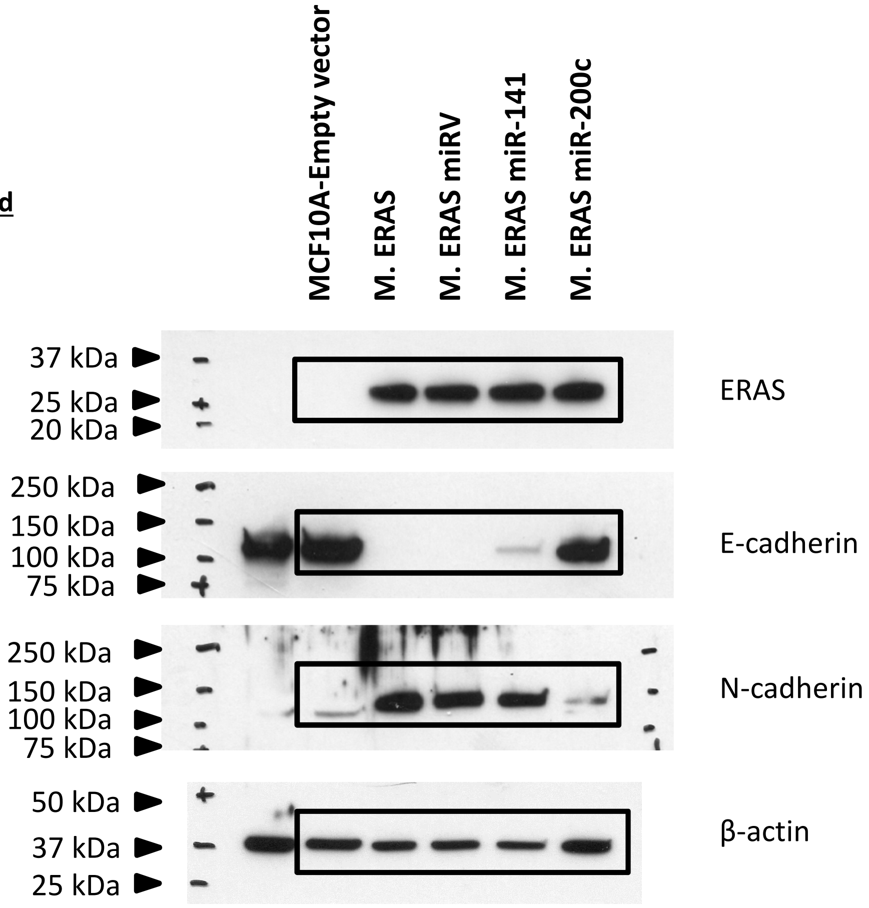

Supplementary Figure S6 (cont.)

Original blots for Fig. 7d and supplementary Fig. 3b

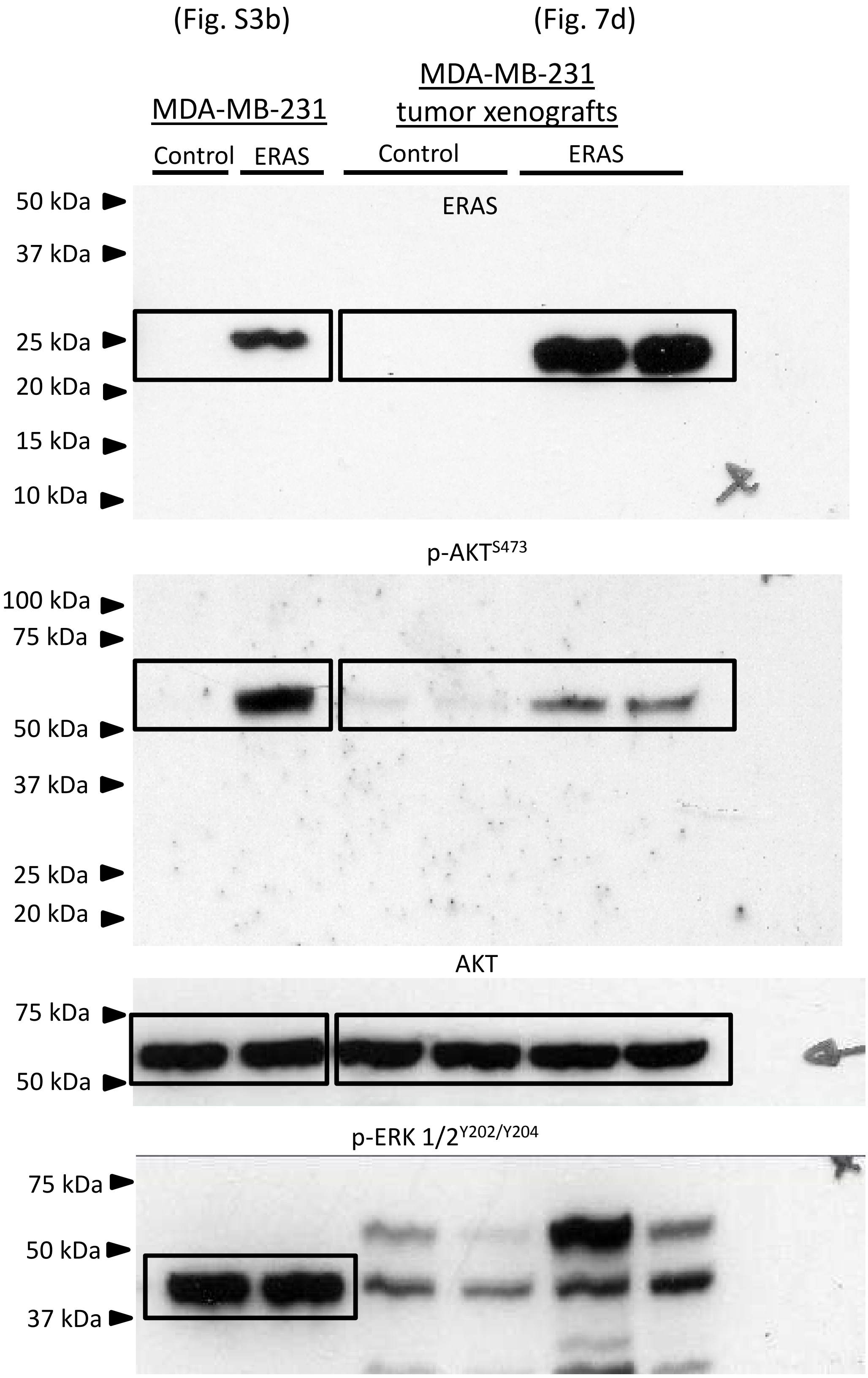

Supplementary Figure S6 (cont.)

Original blots for Fig. 7d and supplementary Fig. 3b (continued)

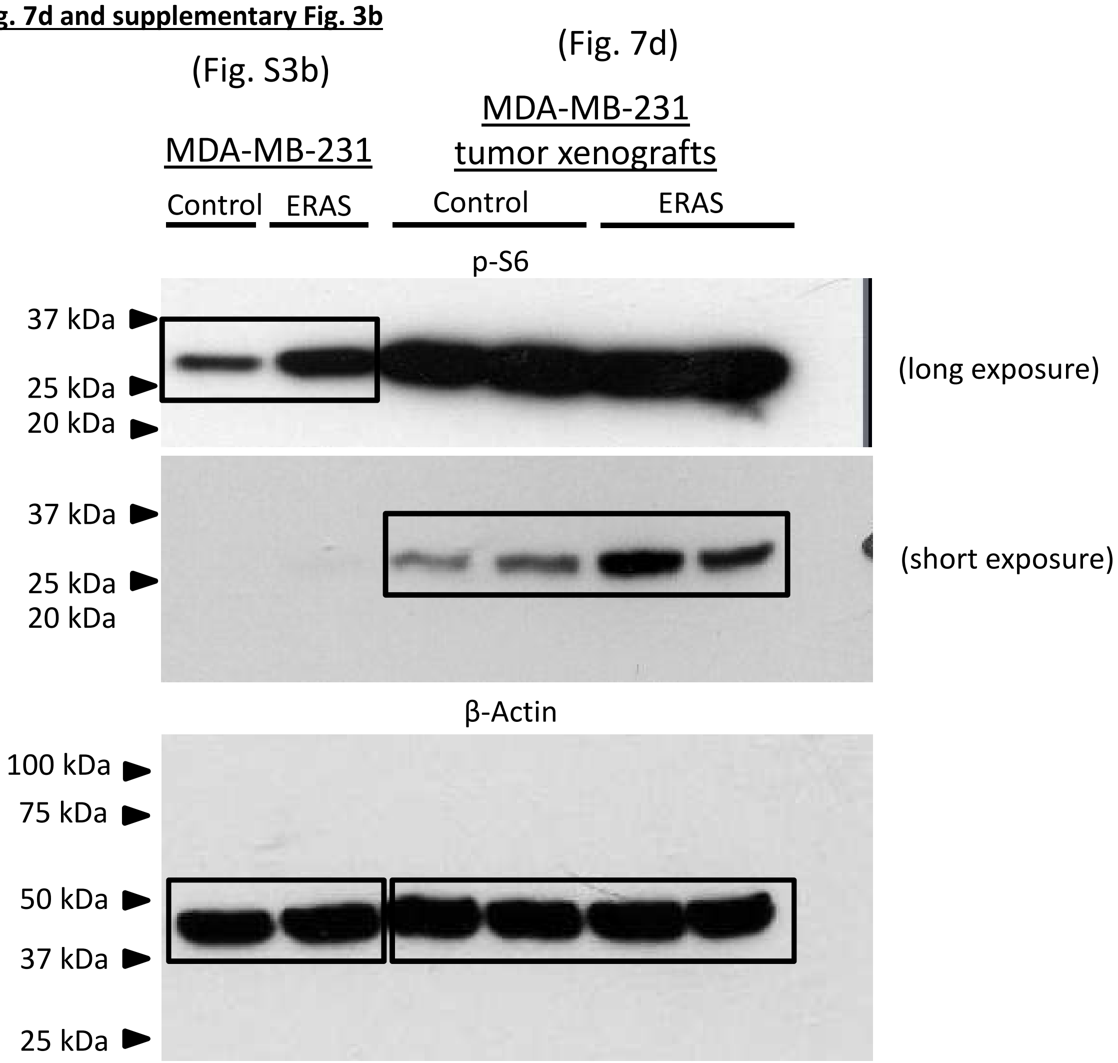

Original blots for supplementary Fig. 3b (continued)

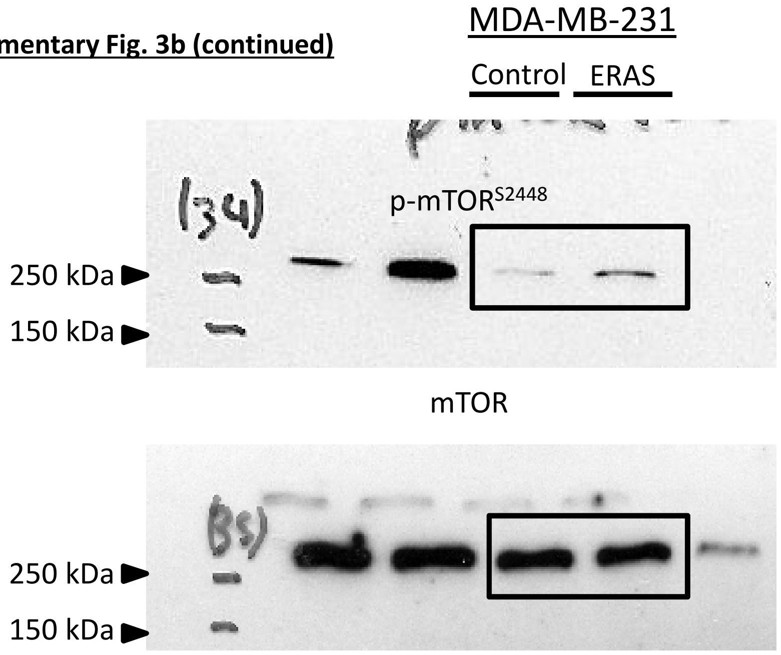

Supplementary Figure S6 (cont.)
